# Unified contact layer and low-temperature transient liquid phase interconnection for high-performance all-Mg-based thermoelectric devices

**DOI:** 10.1093/nsr/nwaf227

**Published:** 2025-05-31

**Authors:** Shanghao Chen, Tianyu Zhang, Jinxuan Cheng, Baopeng Ma, Xiaojing Ma, Xiaofang Li, Li Yin, Linmao Wen, Jun Mao, Feng Cao, Qian Zhang

**Affiliations:** National Key Laboratory of Precision Welding & Joining of Materials and Structures, Harbin Institute of Technology, Harbin 150001, China; School of Materials Science and Engineering, and Institute of Materials Genome & Big Data, Harbin Institute of Technology, Shenzhen 518055, China; School of Materials Science and Engineering, and Institute of Materials Genome & Big Data, Harbin Institute of Technology, Shenzhen 518055, China; School of Materials Science and Engineering, and Institute of Materials Genome & Big Data, Harbin Institute of Technology, Shenzhen 518055, China; School of Materials Science and Engineering, and Institute of Materials Genome & Big Data, Harbin Institute of Technology, Shenzhen 518055, China; School of Materials Science and Engineering, and Institute of Materials Genome & Big Data, Harbin Institute of Technology, Shenzhen 518055, China; School of Materials Science and Engineering, and Institute of Materials Genome & Big Data, Harbin Institute of Technology, Shenzhen 518055, China; School of Science, Harbin Institute of Technology, Shenzhen 518055, China; School of Materials Science and Engineering, and Institute of Materials Genome & Big Data, Harbin Institute of Technology, Shenzhen 518055, China; National Key Laboratory of Precision Welding & Joining of Materials and Structures, Harbin Institute of Technology, Harbin 150001, China; School of Materials Science and Engineering, and Institute of Materials Genome & Big Data, Harbin Institute of Technology, Shenzhen 518055, China; School of Science, Harbin Institute of Technology, Shenzhen 518055, China; National Key Laboratory of Precision Welding & Joining of Materials and Structures, Harbin Institute of Technology, Harbin 150001, China; School of Materials Science and Engineering, and Institute of Materials Genome & Big Data, Harbin Institute of Technology, Shenzhen 518055, China

**Keywords:** low-grade waste heat recovery, all-Mg-based thermoelectric devices, contact layer, transient liquid phase bonding

## Abstract

Thermoelectric technology enables direct conversion of untapped low-grade waste heat into electrical energy. Mg₃(Sb, Bi)₂ and MgAgSb, with their excellent thermoelectric performance near room temperature, have emerged as cost-effective and environmentally friendly alternatives to Bi₂Te₃-based materials. However, the development of high-performance Mg-based thermoelectric devices faces significant challenges due to the inherent high chemical reactivity and volatility of Mg elements, coupled with the phase transition-induced degradation of thermoelectric properties in MgAgSb, which collectively led to poor interfacial contacts and device integration. In this study, a Mg-based thermoelectric device consisting of n-type Mg₃(Sb, Bi)₂ and p-type MgAgSb has been fabricated with Mg₂Ni as the unified contact layer for both materials. The Ni-Sn transient liquid-phase (TLP) low-temperature bonding technology has been employed for the integration of the thermoelectric device. In addition, thermal aging and cycling tests confirmed the long-term stability of the Mg₂Ni/TE contact interfaces and the Ni-Sn intermetallic compound (IMC) joints. Notably, the device with segmented n-type legs achieves an exceptional conversion efficiency of ∼10.8% at a temperature difference of 300 K. This work promotes the application of high-performance, environmentally friendly Mg-based thermoelectric devices in low-grade waste heat recovery.

## Teaser

Breakthrough in eco-friendly Mg-based thermoelectric devices: stable and high-efficiency energy harvesting from low-grade waste heat through innovative interface design and advanced interconnections.

## INTRODUCTION

In modern industry, a mere one-third of the total energy input is effectively harnessed, with the remaining two-thirds being dissipated as waste heat [1,2]. Most of the waste thermal energy exists in low-grade form (below 573 K), which is frequently discarded into the environment due to its low energy density and the technical difficulties associated with its recovery [3]. Efficient utilization of this energy is crucial for improving efficiency and reducing carbon emissions [4,5]. Thermoelectric (TE) technology offers a simple, clean, and noise-free way to directly convert waste heat into electricity [6,7], making it a promising solution for low-grade waste heat recovery [8,9].

Among various TE materials explored to date, Bi₂Te₃ remains the only commercially viable material for refrigeration and power generation [10–13]. However, its large-scale deployment is constrained by the scarcity of elemental tellurium and the pronounced bipolar effect that significantly degrades performance above 450 K [14,15]. These challenges have driven extensive research into the exploration and development of alternative high-performance thermoelectric materials for near-room-temperature applications. In recent years, Mg-based thermoelectric materials, such as n-type Mg_3_(Sb, Bi)_2_ and p-type MgAgSb, have achieved a dimensionless figure-of-merit (*zT*) comparable to that of Bi₂Te₃-based materials around room temperature [16–21]. Beyond demonstrating exceptional thermoelectric performance, these materials emerge as competitive candidates for replacing conventional Bi₂Te₃ alloys [22] through their combined advantages of environmental friendliness, cost-effectiveness, and elemental abundance.

For the practical implementation of thermoelectric technology in a low-grade waste heat recovery scenario, the integration of high-performance TE materials into devices is crucial [23]. The fundamental challenges at the device level primarily originated from the contact layer design and device interconnection, both are crucial in simultaneously minimizing the electrical and thermal parasitic losses, alleviating thermal stress, and consequently enhancing the performance and reliability of TE devices [24]. Extensive investigations have been conducted on thermoelectric interfacial contact design and interconnection technique optimization [25–27]. Traditionally, the development of a thermoelectric contact layer usually relies on tedious trial-and-error experiments, and identifying reliable contact layer materials for both n-type and p-type thermoelectric elements further complicates the process [28–30]. Given the compositional similarities between Mg₃(Sb, Bi)₂ and MgAgSb, there is theoretical potential for a unified thermoelectric interface material. However, no studies have yet demonstrated the simultaneous use of a single alloy as a contact layer for both Mg₃(Sb, Bi)₂ and MgAgSb. In addition, considering the phase transition of MgAgSb at 573 K leads to severe deterioration of its thermoelectric properties, maintaining the soldering temperature of Mg-based devices below this critical threshold is of great significance [31,32]. Nevertheless, to prevent creep deformation in solder bonding layers, thermoelectric devices are typically soldered at temperatures above their operational temperatures, often exceeding 200 K [33]. This contradiction poses a great challenge for achieving reliable integration of high-performance and long-term stable Mg-based thermoelectric devices [34,35].

In this work, Mg_2_Ni is selected as a unified contact layer for both Mg₃(Sb, Bi)₂ and MgAgSb, providing a robust interlayer contact and optimal coefficient of thermal expansion (CTE) matching between the n-type and p-type thermoelectric legs. Furthermore, Ni-Sn transient liquid phase (TLP) bonding is employed to achieve device integration at low temperature [36]. The soldered joints maintain excellent electrical conductivity and shear strength even after isothermal aging at 573 K for 1440 hours. Based on these advancements, an all-Mg-based thermoelectric device with p-type MgAgSb and segmented n-type Mg₃(Sb, Bi)₂ is successfully fabricated, achieving a conversion efficiency of 10.8% at a temperature difference of 300 K. Additionally, the device demonstrates long-term stability below the phase transition temperature of MgAgSb (573 K). These results signify the potential of Mg-based thermoelectric devices as high-performance and sustainable alternatives to traditional Bi₂Te₃ systems, advancing the application of thermoelectric power generation for low-grade waste heat recovery.

## RESULT AND DISCUSSION

### Design of the all-Mg-based thermoelectric device

**Figure 1.**
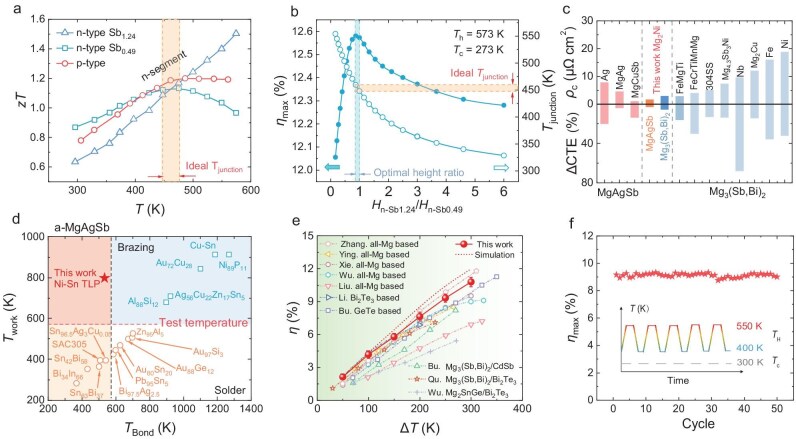
Design, fabrication, and characterization of a Mg-based thermoelectric device. (a) Temperature-dependent *zT* of the fabricated materials; n-type Sb_0.49_: Mg_3.15_ Mn_0.05_Sb_0.49_Bi_1.50_Se_0.01_, n-type Sb_1.24_: Mg_3.15_Mn_0.05_Sb_1.24_Bi_0.75_Se_0.01_, p-type: MgAg_0.965_Ni_0.005_Sb_0.99_. (b) Simulation of the segmented n-type single-leg device. Maximum conversion efficiency *η*_max_ (solid curve) and the junction temperature *T*_junction_ between n-Sb_0.49_ and n-Sb_1.24_ (hollow curve) as a function of the height ratio of *H*_n-Sb1.24_/*H*_n-Sb0.49_ for segmented single leg. (c) The *ρ*_c_ and ∆CTE for this connection design in comparison with those from other reports. [29,39,41–47]. (d) The relationship of working temperature *T*_work_ and connecting temperature *T*_bond_ of some solders and the Ni-Sn TLP bonding. (e) Maximum conversion efficiency (*η*_max_) as a function of hot-side temperature for the two-pair device. Literature data from other devices in low-grade waste heat temperature range are included [19,22,39,48–54]. (f) *η*_max_ of the Mg-based device throughout thermal cycling between hot-side temperatures of 400 K and 550 K. Inset: schematic illustration of the thermal cycling over time.

Herein, MgAg_0.965_Ni_0.005_Sb_0.99_ is chosen as the p-type leg, Ni-doping helps this composition achieve an average *zT* value of ∼1.08 within the temperature range of 293–573 K. Moreover, within a specific doping range, the Ni dopant concentration does not induce significant fluctuations in the *zT* value of MgAgSb (Fig. S3), which indicates that trace amounts of Ni diffusion at the interface will not adversely affect the whole TE properties of the p-type materials. Meanwhile, by adjusting the ratio of Mg vacancies and Bi alloying in the n-type materials, Mg_3.15_Mn_0.05_Sb_0.49_Bi_1.50_Se_0.01_ (n-Sb_0.49_) and Mg_3.15_Mn_0.05_Sb_1.24_Bi_0.75_Se_0.01_ (n-Sb_1.24_) achieved average *zT* values of 1.08 and 1.33, respectively, in the temperature ranges of 293–448 K and 474–574 K. Detailed TE properties are presented in Figs S1–S3. Based on the temperature dependence of *zT* for these materials (Fig. 1a), n-Sb_1.24_ and n-Sb_0.49_ are selected as the high- and low-temperature sides, respectively, to design the segmented n-type legs [37]. To maximize the average *zT* of the n-type leg, the junction temperature (*T*_junction_) at the interface between the high- and low-temperature sections is targeted within the range of 448–474 K, where the *zT* curves of the constituent materials intersect. Finite element simulations have been conducted to evaluate the leg geometry (height ratio of n-Sb_1.24_ to n-Sb_0.49_) on the device performance at hot-side temperatures (*T*_hot_) of 573 K and cold-side temperatures (*T*_cold_) of 273 K (Fig. S5). The optimal ratio of *H*_n-Sb0.49_/*H*_n-Sb1.24_ is determined to be in the range of 0.8–1.0 (Fig. 1b). This ratio achieves an ideal *T*_junction_ of ∼450 K and is predicted to yield a maximum conversion efficiency of ∼12.6% for the n-type segmented leg.

Extensive research has been conducted on thermoelectric interface materials (TEiMs) of n-type Mg_3_(Sb, Bi)_2_. In our previous work, Mg_2_Ni was successfully screened out as an excellent contact material for Mg_3_(Sb, Bi)_2_ through the calculation of phase diagram (CALPHAD) [38]. The similarity in the chemical compositions between Mg_3_(Sb, Bi)_2_ and MgAgSb suggests the possibility of developing a single interface material capable of serving both n-type and p-type Mg-based TE legs. Additionally, it was reported that both Mg and Ni can be effectively bonded to MgAgSb [39], exhibiting passivated diffusion and lower contact resistance compared to other pure metals. However, the Mg contact layer suffers from poor long-term stability, whereas the Ni contact layer demonstrates inadequate densification during low-temperature sintering (below 573 K). To address these issues, we developed Mg_2_Ni as a low-melting-point TEiM for MgAgSb, and the Mg-rich environment at the contact interface can effectively mitigate the formation of Ag₃Sb and Sb phases [40]. Remarkably, the contact resistivity (*ρ*_c_) and thermal expansion coefficient mismatch (ΔCTE) between Mg_2_Ni/MgAgSb and Mg_2_Ni/Mg₃(Sb, Bi)₂ have been significantly reduced compared to the previous reports (Fig. 1c). This improvement establishes optimal compatibility in both electrical and mechanical properties of the thermoelectric legs, thereby ensuring high performance and reliability of the thermoelectric device.

To avoid the phase transformation in MgAgSb above 573 K and to fully exploit the potential of Mg-based devices in low-grade waste heat power generation, an interconnection technology is required that allows welding below 573 K while ensuring long-term stability at elevated temperatures. The common low-temperature solders (the orange hollow circle) cannot guarantee the proper operation of Mg-based devices at maximum temperature differences. In contrast, the working temperature (*T*_work_) of traditional fillers depends linearly on their bonding temperature (*T*_bond_), following the relation *T*_work_ = 0.8 × (*T*_bond_ − 50 K). This relationship indicates that the bonding temperature of traditional fillers exceeds their working temperature, and the brazing process (the blue hollow square) will inevitably trigger phase transformations in MgAgSb, ultimately resulting in device failure (Fig. 1d). As an advanced interconnection technology, transient liquid phase (TLP) bonding employs a low-melting-point interlayer to create a transient liquid phase, promoting atomic diffusion at relatively low temperatures and pressures then subsequently forming a high-melting-point solid bonding layer. Furthermore, based on the diverse joining systems, appropriate filler alloys can be selected to achieve effective bonding with various electrodes and contact layers, which enhances the versatility of TLP bonding in fabricating thermoelectric devices. Given these considerations, we used SAC305 (*T*_bond_ = 490 K) for TLP bonding between the Cu electrode covered with an electroplated Ni layer and Ni metallization layers, forming high-melting-point Ni-Sn intermetallic compounds (Ni-Sn IMCs).

Finally, we successfully constructed two pairs of segmented Mg-based devices, achieving an impressive thermoelectric conversion efficiency of 10.8% at a temperature difference of 300 K, which is essentially comparable to the current state-of-the-art device under similar conditions (Fig. 1e). The thermodynamic stability of the contact layer and the bonding layer ensures the exceptional long-term stability of the devices. To assess the thermal stability and operational reliability of the device, 50 consecutive thermal cycling tests between 400 K and 550 K have been performed to monitor the conversion efficiency and output power under controlled temperature differences. Notably, the device maintains superior stability in output power and conversion efficiency during cycling, highlighting its robustness and reliability under varying thermal conditions (Fig. 1f).

### Enhanced stability of interfaces using a unified Mg₂Ni alloy

**Figure 2.**
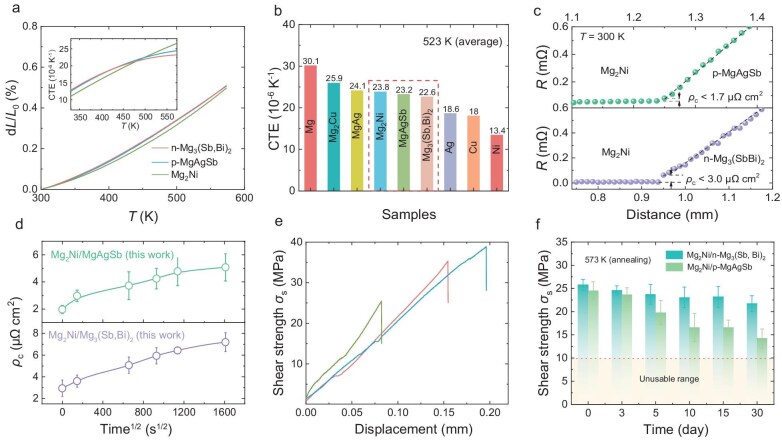
The electrical and mechanical compatibility of Mg_2_Ni alloy with MgAgSb and Mg₃(Sb, Bi)₂. (a) d*L*/*L*_0_ versus temperature curve for Mg_2_Ni, Mg₃(Sb, Bi)₂ and MgAgSb. (b) Coefficients of thermal expansion of Mg_2_Ni, Mg₃(Sb, Bi)₂, and MgAgSb compared with that of common electrode metals and their alloys [28,39,58]. (c) Measured contact resistivity (*ρ*_c_) of Mg_2_Ni/Mg₃(Sb, Bi)₂ and Mg_2_Ni/MgAgSb junctions. (d) The aging time dependent of the contact resistivity for the Mg_2_Ni/Mg₃(Sb, Bi)₂ and Mg_2_Ni/MgAgSb junctions aged at 573 K (specific contact resistance values for different aging times are shown in Figs S14 and S15). (e) Change in *σ*_s_ vs displacement curve of Mg_2_Ni, Mg₃(Sb, Bi)₂, and MgAgSb for estimating the *σ*_s_. (f) The shear strength of Mg_2_Ni/Mg₃(Sb, Bi)₂ and Mg_2_Ni/MgAgSb junctions aged at 573 K under 0–30 days aging time.

As shown in Fig. 2a, the d*L*/*L*_0_ of Mg₂Ni is close to that of both n-type and p-type thermoelectric materials within the temperature range of 293–573 K. The CTE of Mg₂Ni (23.8 × 10^−^^6^ K^−^^1^) closely matches the n-type (22.6 × 10^−^^6^ K^−^^1^) and p-type (23.3 × 10^−^^6^ K^−^^1^) TE materials compared to other contact layers (Fig. 2b). This exceptional CTE match can effectively minimize interfacial stresses during thermal cycling, thereby enhancing the mechanical integrity and reliability of the thermoelectric device.

The electrical and mechanical compatibility of Mg₂Ni/MgAgSb and Mg₂Ni/Mg₃(Sb, Bi)₂ joints have been systematically evaluated. As shown in Fig. 2c, the initial contact resistivities of Mg₂Ni/Mg₃(Sb, Bi)₂ and Mg₂Ni/MgAgSb junctions were measured at 3.0 μΩ·cm² and 1.7 μΩ·cm², respectively. The distribution of elements and the contact resistance scan of the whole leg are shown in Figs S9–S11. Given that Mg₃(Sb, Bi)₂ is a highly degenerate semiconductor [55] and MgAgSb is a semi-metal [56], and assuming minimal interfacial chemical reactions, these interfaces behave more like metal-to-metal contacts rather than conventional semiconductor-to-metal contacts. This characteristic implies a high density of states near the Fermi level, facilitating carrier transport across the interface with minimal potential barriers [57], and resulting in ultralow contact resistivity. To assess the thermal stability of the interface, accelerated aging tests were conducted at 573 K. The interfacial integrity between Mg_2_Ni/Mg₃(Sb, Bi)₂ and Mg_2_Ni/MgAgSb maintains exceptional stability without observable microcracks or delamination (Figs S12 and S13). Additionally, no significant elemental diffusion was detected, and the interfacial reaction layer remained minimal. Figures S14 and S15 show the linear scanning of resistance across the junctions of Mg₂Ni/Mg₃(Sb, Bi)₂ and Mg₂Ni/MgAgSb at different aging durations. The resistivity rose slowly to 7.2 ${\mathrm{\mu \Omega }}$ cm^2^ and 5.1 ${\mathrm{\mu \Omega }}$ cm^2^ for Mg_2_Ni/Mg₃(Sb, Bi)₂ and Mg_2_Ni/MgAgSb joints, respectively, after aging for 720 h (Fig. 2d).

Additionally, we evaluated the shear strength of two joints, together with that of Mg₂Ni, MgAgSb, and Mg₃(Sb, Bi)₂. As shown in Fig. 2e, the shear strength (*σ*_s_) of Mg₂Ni is 25.5 MPa, which is lower than that of Mg₃(Sb, Bi)₂ (35.3 MPa) and MgAgSb (38.8 MPa). The initial shear strength of Mg₂Ni/Mg₃(Sb, Bi)₂ and Mg₂Ni/MgAgSb joints was ∼25 MPa, demonstrating satisfactory performance. Benefiting from the matched coefficients of thermal expansion and stable thermodynamic state, the shear strength of Mg₂Ni/Mg₃(Sb, Bi)₂ junctions decays very slowly during long-term aging at 573 K. In contrast, Mg_2_Ni/MgAgSb junctions exhibit some degradation, possibly due to the weaker diffusion and reaction elements at Mg_2_Ni/MgAgSb interfaces annealed at 573 K. Nevertheless, the junction strength remains well above 10 MPa (Fig. 2f), ensuring long-term reliability and performance.

### Low-temperature Ni-Sn TLP connection for all-Mg-based thermoelectric device

**Figure 3.**
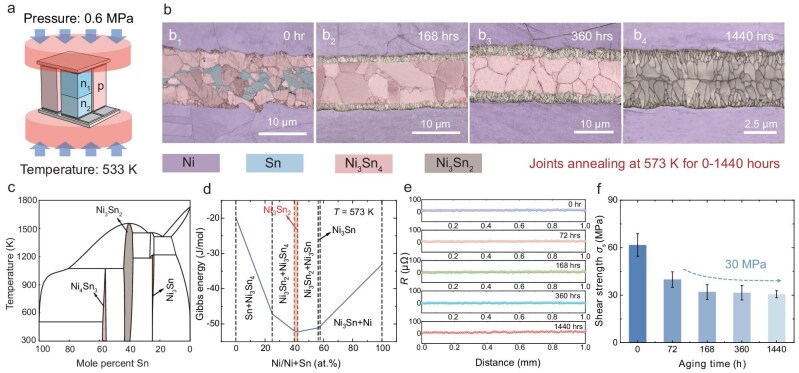
Characterization of the Ni-Sn TLPB connection layer throughout the isothermal aging duration at 573 K. (a) Schematic diagram showing the assembly of the thermoelectric device by Ni-Sn transient liquid phase bonding. (b) Phase maps overlapped with grain boundaries after aging for different times (b_1_-b_4_: 0, 168, 360, and 1440 hours). (c) Possible phases and transformation processes formed in the phase diagram of Ni-Sn TLP joints [63]. (d) Calculated Gibbs free energy for Ni-Sn joints at 573 K. (e) The variation of the contact resistance of joints with increasing aging time. (f) The variation of the shear strength of joints with increasing aging time.

Once the soldering temperature exceeds the melting point of Sn (505 K), Ni atoms can easily dissolve into the liquid Sn at the interface and transform into solid Ni-Sn intermetallic compounds (Ni-Sn IMC) during the subsequent isothermal solidification process, consequently Ni-Sn TLP joints can be achieved at lower temperatures and pressures. Figure 3a illustrates the TLP sintering process used in this work, which is conducted at a temperature of 533 K and a pressure of 0.6 MPa. In our previous study, TLP technology successfully achieved effective connections in medium-temperature GeTe thermoelectric devices [59]. However, the microstructure and performance of Ni-Sn TLP joints are highly dependent on the isothermal diffusion kinetics and IMC growth at operational temperatures [60]. In other words, joint performance can vary significantly with different operational temperatures and durations, thereby a thorough understanding of the isothermal diffusion and growth kinetics of Ni-Sn IMC joints at 573 K is crucial.

A key focus in the study of Ni-Sn TLP diffusion kinetics is the type of IMC formed during the interfacial reaction. Based on the Ni-Sn binary phase diagram (Fig. 3c), the progressive interdiffusion of Ni and Sn atoms induces sequential phase transformations, initiating with the formation of Ni₃Sn_4_, followed by the transition to Ni₃Sn₂, and culminating in Ni₃Sn as the Sn solder undergoes complete consumption [61]. Due to the constrained dimensions of the welding interface and its intimate integration with the adjacent Ni substrate, precise phase identification for the specific IMCs through conventional X-ray diffraction (XRD) analysis presents technical challenges. To address this issue, electron backscatter diffraction (EBSD) analysis has been employed to reveal the specific phase evolution of Ni-Sn joints during the isothermal aging process at 573 K. Figure 3b shows overlaid images of phase mappings and diffraction patterns from the joint during isothermal aging. Notably, different shades within the same phase represent varying grain orientations (Fig. S16). The experimental results indicate that the as-sintered welding interface primarily consists of the single Ni₃Sn₄ phase along with the residual Sn solder. With the progressive annealing time, the interfacial welding zone undergoes continuous narrowing, accompanied by the gradual depletion of residual Sn solder. A secondary phase Ni_3_Sn_2_ begins to nucleate and grow inward from the interface. The growth of Ni_3_Sn_2_, along with the depletion of the original welding zone, proceeds at an exceedingly slow rate. Ultimately, the final morphology of the weld stabilizes into single-phase Ni_3_Sn_2_ and remains unchanged even after 1440 hours of annealing, indicating its long-term stability. This result aligns perfectly with the combined backscattered electron (BSE) characterization and the elemental analysis in Fig. S17 and Table S4, providing a clear and quantitative insight into the phase evolution of Ni-Sn IMCs under these conditions.

Nonetheless, based on the phase diagram, the theoretically predicted final intermetallic compound (IMC) should be Ni₃Sn. However, our experimental results show that the transformation process halts at Ni₃Sn₂. To further elucidate the thermodynamics of IMC phase transformations, we calculated the Gibbs free energies for Ni-Sn phase transitions (Fig. 3d). During aging at 573 K, as the Sn ratio decreases, the interfacial phases are expected to evolve through the sequence of Sn + Ni₃Sn₄, Ni₃Sn₄, Ni₃Sn₄ + Ni₃Sn₂, Ni₃Sn₂ + Ni₃Sn, Ni₃Sn, and Ni₃Sn + Ni. Thermodynamic analysis reveals that Ni₃Sn₂ possesses minimal Gibbs free energy across all potential interfacial configurations [62], demonstrating its thermodynamic stability as the equilibrium phase. In other words, the thermodynamic barrier prevents further transformation from Ni₃Sn₂ to Ni₃Sn under these aging conditions.

After gaining a clear understanding of the phase transformation behavior in the welding joints, it is essential to evaluate their electrical and mechanical performance to assess the feasibility of the Ni-Sn IMC joints for long-term operation in thermoelectric devices. The observed IMC exhibits minimal internal defects, which is beneficial for the electrical performance of the joint. Contact resistance measurement indicates that the parasitic electrical losses are negligible at the interface even after annealing for more than 1440 hours (Fig. 3e and Fig. S18), indicating excellent reliability during the operational lifetime of thermoelectric devices. To further assess the interfacial mechanical properties, shear strength measurements were conducted on joints with varying aging conditions. The results reveal that the as-welded joints exhibit the highest shear strength of ∼60 MPa (Fig. 3f: before aging), likely due to the relatively ductile nature of residual Sn solder [59]. Extended annealing processes lead to the complete dissolution of the residual Sn solder, resulting in weld joints composed entirely of Ni-Sn IMCs. Consequently, owing to the intrinsic brittleness of IMCs, the interfacial shear strength of the fully transformed IMC joints is ∼30 MPa (Fig. 3f: after aging for 168 hours), maintaining this mechanical stability despite subsequent phase evolution. Such strength of the IMC joints remains significantly higher than that of the contact interfaces between the TEiM and TE materials, ensuring excellent mechanical integrity under operational conditions.

### High performance of all-Mg-based thermoelectric device

By using Mg₂Ni as the unified contact layer for both n-type and p-type materials, and leveraging the TLP low-temperature joining technology, we fabricated two pairs of devices composed of Mg_3.15_Mn_0.05_Sb_1.24_Bi_0.75_Se_0.01_ and MgAg_0.965_Ni_0.005_Sb_0.99_. The simulated and measured performance of the devices are presented in Figs S19 and S20. Benefitting from the excellent compatibility between Mg₂Ni and the TE materials, combined with the optimized interfacial bonding enabled by the TLP process, a maximum conversion efficiency of 9.1% was achieved under a temperature difference of 300 K (the theoretical efficiency is ∼10.3%). The simulations presented in Fig. S21, indicate that optimizing the electrical contact resistance is crucial for enhancing conversion efficiency. This result is comparable to that of the state-of-the-art Mg-based devices [39,42,64], and surpasses the commercial Bi₂Te₃-based devices.

**Figure 4.**
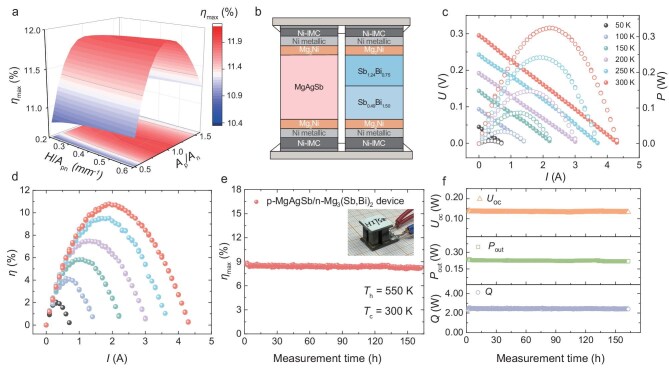
Performance of the two-pairs segmented all-Mg-based device. (a) Simulated *η*_max_ as a function of the cross-sectional area ratio of the p- and n-type legs (*A*_p_/*A*_n_) and the ratio of height to the total cross-sectional area of the legs (*H*/*A*_pn_). (b) Schematic of Mg-based device structure design, including thermoelectric legs, barrier layers, solder layers (Ni-IMC), electrode layers, and pre-circuited AlN ceramic plates. (c and d) Measured voltage (*U*), output power (*P*) and efficiency (*η*) as a function of current (*I*) at different temperatures. (e and f) *η*_max_, open circuit voltage (*U*_oc_), maximum output power (*P*_out_), heat flow (*Q*) of the device at a Δ*T* of 250 K during 160-hour isothermal aging test.

To further improve the performance of the device, the segmented n-type legs were designed, and the influence of leg geometry was investigated. Generally, the temperature difference across the TE legs elevates when the length of the TE legs increases, this will enhance the conversion efficiency while compromising the power density at a fixed temperature difference. In addition, a cross-sectional area ratio *A*_p_/*A*_n_ close to unity ensures uniform heat transfer and thermal stress distribution. Using finite element simulations (Fig. 4a), we determined that the *A*_p_/*A*_n_ has a salient impact on efficiency at a fixed temperature difference, while an *A*_p_/*A*_n_ ratio between 0.8 and 1 maximizes efficiency. To simplify manufacturing and ensure adequate output power, all legs were designed with dimensions of 3.5 × 3.5 × 7 mm³. Furthermore, contact resistivity at the interface of n-Sb_0.49_/n-Sb_1.24_ is only ∼1.9 ${\mathrm{\mu \Omega }}$ cm^2^, and elemental diffusion is not observable (Fig. S22), confirming the feasibility of the segmented design. Therefore, we constructed thermoelectric devices with a multilayer structure including TE legs (segmented n-type legs), Mg_2_Ni barrier layer, solder layer (IMC layer obtained by Ni-Sn TLP soldering), electrode layer, and AlN ceramic plate (Fig. 4b).

Figure 4c and d displays the output voltage (*U*), output power (*P*), and thermoelectric conversion efficiency (*η*) versus current (*I*) for the two-pair segmented Mg-based device under varying temperature differences. To prevent phase transformation in MgAgSb, a temperature difference Δ*T* of 300 K was achieved by fixing the cold side temperature (*T*_c_) at 273 K. For measurements at alternative temperature differences, *T*_c_ was maintained at 293 K. The good linearity of the *I*–*V* curve guarantees the accurate evaluation of the open-circuit voltage (*U*_oc_) and internal resistance (*R*_in_) of the device. The measured *U*_oc_ closely matches the calculated value (Fig. S23), while *R_in_*, derived from the slope of the *I*–*V* curve (Fig. S24), shows minimal deviations at lower temperatures and achieves convergence as the temperature increases. As a result, the all-Mg-based device demonstrates a power density of nearly 0.32 W cm⁻² as well as achieving an impressive conversion efficiency of ∼10.8% under a Δ*T* of 300 K (Fig. 4d), representing an 18% improvement over the single-stage device. The experimental measurements demonstrate good agreement with simulations in terms of the electrical output performance. However, a discrepancy is noted between the measured heat flux and simulations at higher temperatures (Fig. S25), which can be attributed to thermal radiation. It should be highlighted that quantifying the radiation dissipation and its impact on measured heat flow is challenging.

It is essential to emphasize that achieving high *η* is not the only goal, the durability of TE devices is equally important for practical applications. To evaluate the operational stability of the device, we conducted aging tests on the devices at *T*_h_ = 550 K. Figure 4e demonstrates the stable operating condition of the device for 160 hours. Throughout this nearly one-week testing period (Fig. 4f), there were no significant fluctuations in the open-circuit voltage (*U*_oc_), output power (*P*_out_), or heat flow (*Q*). As evidenced by SEM analysis (Figs S26 and S27), the hot-end joint exhibits no obvious holes and cracks after 160 hours of service, demonstrating the surface integrity and the rationality of the interface design. Moreover, thermal shock testing results indicate that the device remains robust with no noticeable degradation in conversion efficiency after 50 thermal cycles with Δ*T* ranging from 100 to 250 K. Figure S28 records the normalized results of *U*_oc_, *R*_in_, *Q*_max_, and *η*_max_ during cyclic testing. While some performance changes were observed over the cycles, likely due to the intermittent nature of the testing process, the device consistently exhibited excellent thermal shock resistance and long-term stability.

## CONCLUSION

In conclusion, by innovatively utilizing the unified Mg₂Ni alloy as contact layer in both n-type and p-type legs, as well as leveraging a low-temperature TLP interconnection technology, we successfully developed a high-performance, environmentally friendly Mg-based thermoelectric device. The diffusion-passivated and thermally expansion-matched Mg₂Ni alloy resulted in low contact resistances of 1.7 μΩ·cm² for p-type legs and 3.0 μΩ·cm² for n-type legs, ensuring robust shear strength over extended service periods. By employing Ni-Sn TLP below the phase transition temperature of MgAgSb, we ensured the integrity and excellent thermoelectric performance of the interface. Long-term tests revealed that the final IMC at 573 K is Ni₃Sn₂, validating the electrical and mechanical stability of full IMC joints in thermoelectric devices. Eventually, the Mg-based thermoelectric device with a segmented n-type design achieved a high conversion efficiency of 10.8% under a 300 K temperature gradient, demonstrating outstanding stability and thermal cycling performance. This work underscores the potential of Mg-based thermoelectric devices as a sustainable and cost-effective alternative to Bi₂Te₃, opening new opportunities for thermoelectric technology in low-grade waste heat recovery applications.

## METHODS

### Materials synthesis and device fabrication

For n-type materials, high-purity magnesium particles (Mg, 99.9%), antimony shots (Sb, 99.999%), bismuth shots (Bi, 99.999%), selenium shots (Se, 99.999%), and manganese powder (Mn, 99.9%) were weighted according to the nominal compositions of Mg_3.15_Mn_0.05_Sb_0.49_Bi_1.5_Se_0.01_ and Mg_3.15_Mn_0.05_Sb_1.24_Bi_0.75_Se_0.01_ in a glove box under an argon atmosphere. All the elements were ball-milled for 10 hours, consolidated by spark plasma sintering (SPS) at 1073 K for 5 minutes. For p-type materials, a two-step ball milling process was employed before SPS. Magnesium particles (Mg, 99.9%), silver particles (Ag, 99.999%), and nickel powder (Ni, 99.9%) were first loaded into a stainless steel jar inside an argon-filled glove box and then ball-milled for 10 hours. Subsequently, antimony particles (Sb, 99.99%) were added into the jar under an argon atmosphere for an additional 10-hour ball milling process. The obtained powder was then loaded into a graphite die with an inner diameter of 10 mm and sintered by SPS at 573 K for 30 minutes under a pressure of 85 MPa [65]. XRD results (Fig. S4) indicate that all samples are pure-phase with excellent crystallinity. For Mg_2_Ni, the preparation was carried out by the Mg-Ni phase diagram [66] (Fig. S6). High-purity magnesium particles (Mg, 99.9%) and nickel particles (Ni, 99.9%) were vacuum-sealed in niobium tubes and melted at 1223 K for 12 hours. Excess Mg was added to lower the melting point of the alloy. The resulting ingot was then ball-milled for 1 hour to obtain the thermoelectric interface materials (TEiM) powder, which was then integrated with the TE bulk material via SPS to form the TEiM/TE contact junctions. The X-ray diffraction (XRD) analysis, scanning electron microscopy (SEM) imaging with energy-dispersive spectroscopy (EDS) mapping, and thermal and electrical conductivity measurements of Mg₂Ni alloy sintered at different temperatures are presented in Figs S7 and S8. The geometric optimization of thermoelectric devices was simulated and analyzed using the finite element analysis software COMSOL Multiphysics. The simulation boundary conditions were defined as outlined in Table S1. The Ni/Mg_2_Ni/Mg_3_Sb_2_ junctions were sintered at 673 K under 50 MPa for 10 minutes, while the Ni/Mg_2_Ni/MgAgSb junctions were sintered at 573 K under 85 MPa for 10 minutes to construct the TE legs. All legs were then cut into dimensions of 3.5 × 3.5 × 7 mm³ and bonded to nickel-coated copper ceramic plates using transient liquid phase (TLP) bonding.

## Supplementary Material

nwaf227_Supplementary_File
