# Supplementary material for: Unified contact layer and low-temperature transient liquid phase interconnection for high-performance all-Mg-based thermoelectric devices
Source: Natl Sci Rev. 2025 May 31;12(8):nwaf227. doi: 10.1093/nsr/nwaf227 (PMC12236311; doi:10.1093/nsr/nwaf227)
Supplement: nwaf227_Supplementary_File [file nwaf227_supplementary_file.pdf]

Unified Contact Layer and Low-Temperature Transient Liquid Phase Interconnection for High-Performance All-Mg-Based Thermoelectric Devices

Shanghao Chen[a,b], Tianyu Zhang[b], Jinxuan Cheng[b], Baopeng Ma[b], Xiaojing Ma[b], Xiaofang Li[b], Li Yin[c], Linmao Wen[b], Jun Mao[a,b]*, Feng Cao[c]*, Qian Zhang[a,b]*

[a]*National Key Laboratory of Precision Welding & Joining of Materials and Structures, Harbin Institute of Technology, Harbin 150001, China*

[b]*School of Materials Science and Engineering, and Institute of Materials Genome & Big Data, Harbin Institute of Technology, Shenzhen 518055, China*

[c]*School of Science, Harbin Institute of Technology, Shenzhen 518055, China*

*Corresponding author, email: *maojun@hit.edu.cn*, *caofeng@hit.edu.cn*, *zhangqf@hit.edu.cn*

This supplement file includes

Supplementary methods

Supplementary Figures 1-28

Supplementary Tables 1-7

SUPPLEMENTARY METHODS

Sample characterization

TE propertie

The thermoelectric dimensionless figure-of-merit zT is calculated by $zT = S^2\sigma T/\kappa$, where S is the Seebeck coefficient, σ is the electrical conductivity, and κ is the thermal conductivity. The Seebeck coefficient and electrical conductivity were simultaneously measured using a commercial system (ZEM-3, ULVAC). The thermal conductivity κ was determined via the equation $\kappa = D\rho C_p$, where D represents the bulk density ascertained by the Archimedes method, ρ is the thermal diffusivity obtained using laser flash analysis (LFA 457, Netzsch), and C_p is the specific heat capacity measured by differential scanning calorimetry (DSC 404 F1 Pegasus, Netzsch).

Composition and microstructure

The crystal structures were examined by X-ray diffraction (X'Pert Pro, PANalytical) and electron back-scattered diffraction (EBSD, C NANO)[1]. The interface microstructures were observed by a field emission scanning electron microscopy (FE-SEM, crossbeam 360). The chemical composition of the phases formed at the interface was determined by an energy disperse spectroscopy (EDS) attached on the FE-SEM.

Electrical and mechanical properties

The coefficient of thermal expansion (CTE) was measured by using a thermal mechanical analyzer (TMA 402, Netzsch). The shear strength of each joint was measured using a bonding tester (TRY, MFM-1200). The shear height of the blade tip above the substrate was 100 μm, and the shear speed was 200 μm s^{-1}. A custom-built four-probe measurement system was employed to determine the contact resistivity (ρ_c) using the formula: $\rho_c = A \times R_{jump}$, where the cross-sectional area of each joint is $A = 2.5 \times 2.5$ mm^2, and R_{jump} represents the interval value of resistance at each joint interface.

Device characterization

The output power (P) and conversion efficiency (η) of the TE device under various temperature gradient were measured in a vacuum environment using a homemade

measurement system[2]. Subsequent thermal aging and thermal shock tests were also conducted using this equipment.

Joints fabrication and isothermal aging

Commercially available nickel (Ni) substrates (2 mm thick, 99.9% pure) and SAC305 solder were used (sourced from Advent and Alfa Aesar, respectively). For each sample preparation, a 100 μm thick layer of SAC305 solder was screen-printed onto one Ni substrate, followed by the placement of another Ni substrate to form a sandwich structure. The joints were then bonded at 533 K under a pressure of 0.6 MPa for 10 minutes in air (the detailed heating process is illustrated in Fig. 3a). After bonding, the joints were slowly cooled to room temperature under a reduced pressure of 0.2 MPa[3]. For isothermal aging experiments, all samples were encapsulated in vacuum-sealed quartz tubes and annealed at 573 K for varying durations. It is noteworthy that, in addition to Ni and Sn, the SAC305 solder contains trace amounts of Ag and Cu. Our previous studies have demonstrated that these trace elements of Ag and Cu ultimately dissolve into the Ni-Sn intermetallic compound (IMC)[4]. Therefore, this work focuses exclusively on the phase transformations within the Ni-Sn IMC. The Gibbs free energy in the Ni-Sn binary system was calculated using Thermo-Calc software, with the thermodynamic parameters obtained from the literature[5].

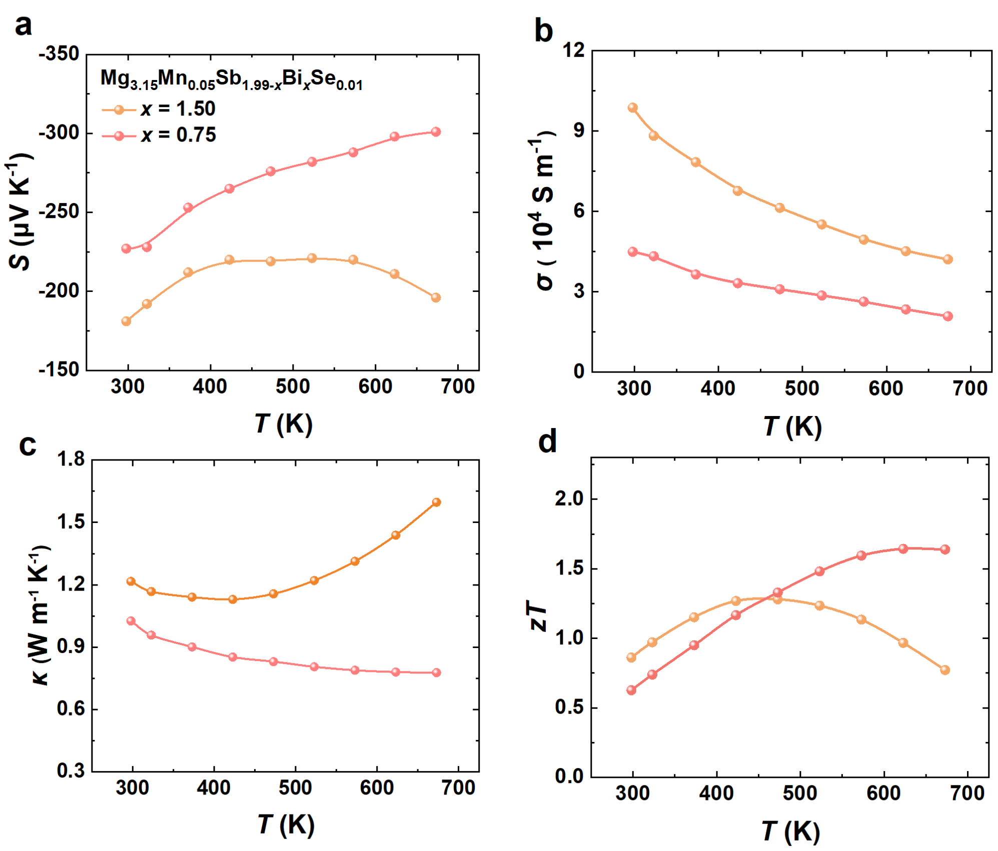

Figure S1. Temperature-dependent (a) Seebeck coefficient, (b) electrical conductivity, (c) total thermal conductivity, and (d) *zT* of n-type $Mg_{3.15}Mn_{0.05}Sb_{1.99\text{-}x}Bi_xSe_{0.01}$ (x = 0.75, 1.50).

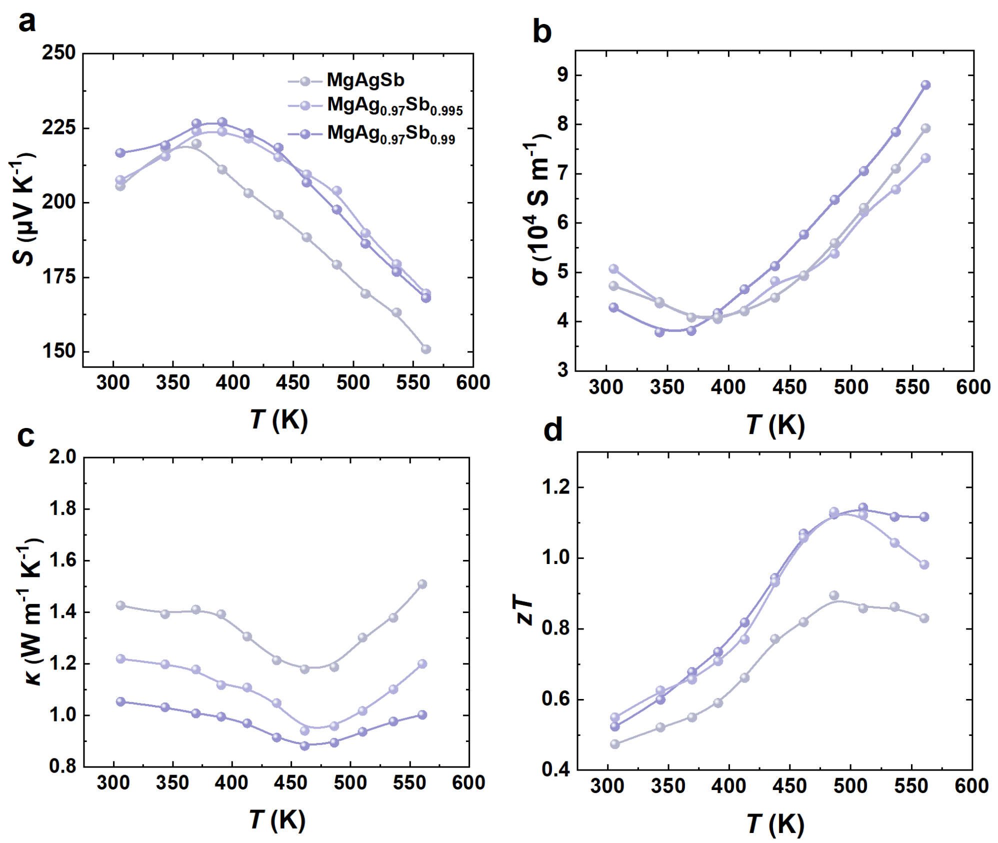

Figure S2. Temperature-dependent (a) Seebeck coefficient, (b) electrical conductivity, (c) total thermal conductivity, and (d) *zT* of p-type MgAgSb.

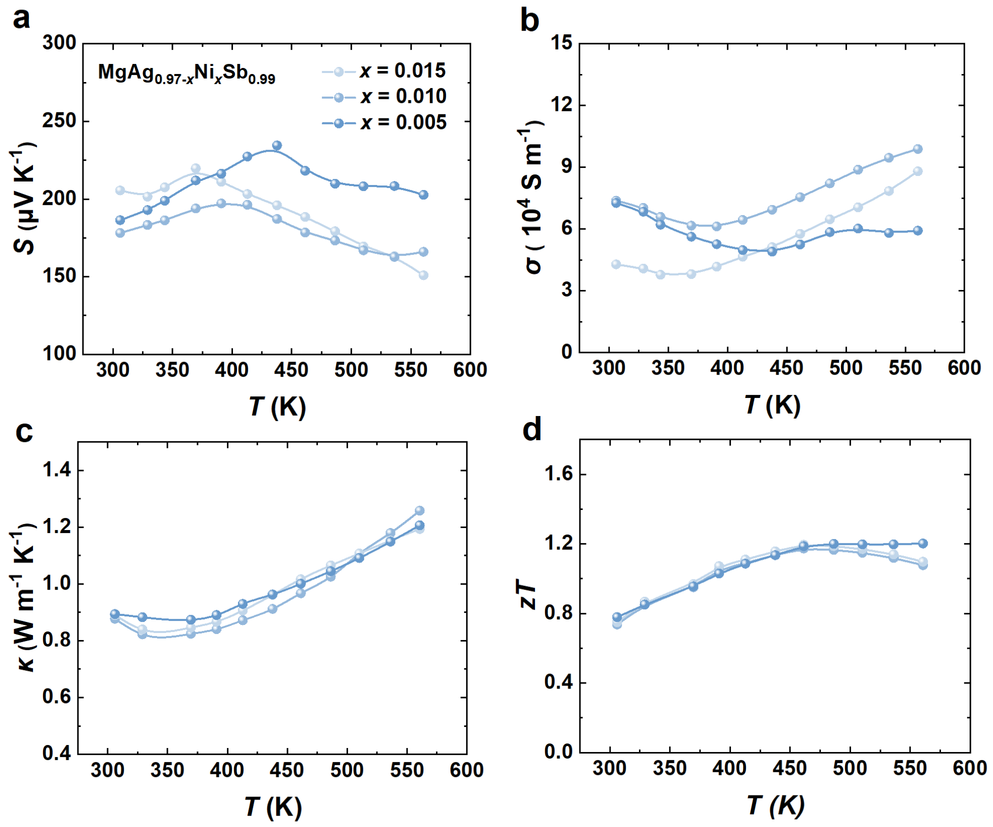

Figure S3. Temperature-dependent (a) Seebeck coefficient, (b) electrical conductivity, (c) total thermal conductivity, and (d) *zT* of Ni doped p-type $MgAg_{0.97-x}Ni_xSb_{0.99}$ (x = 0.05, 0.10, and 0.15).

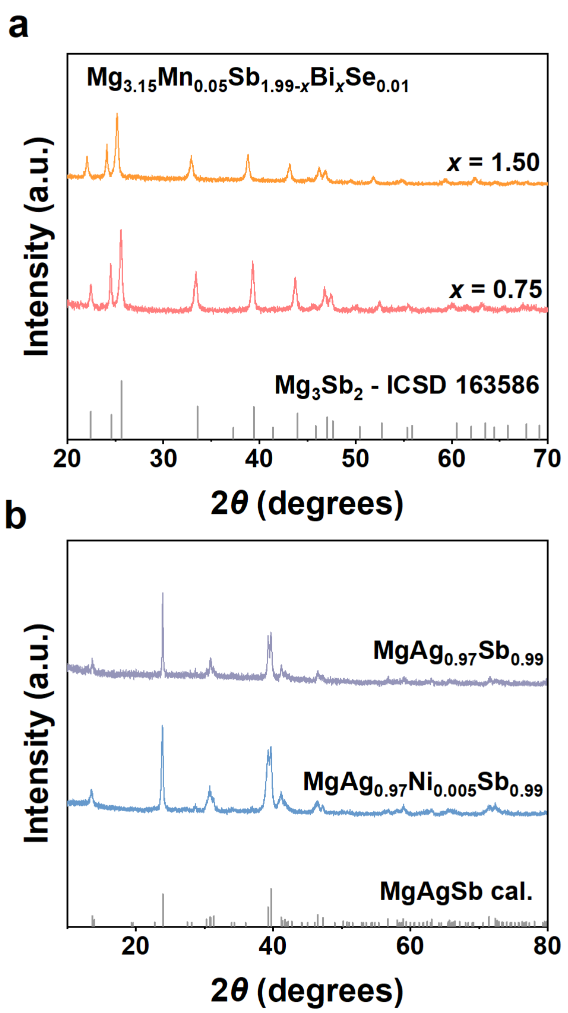

Figure S4. XRD patterns of (a) n-type $Mg_{3.15}Mn_{0.05}Sb_{1.99-x}Bi_xSe_{0.01}$ (x = 0.75, 1.50) and (b) p-type $MgAg_{0.97}Sb_{0.99}$ and $MgAg_{0.965}Ni_{0.005}Sb_{0.99}$[6].

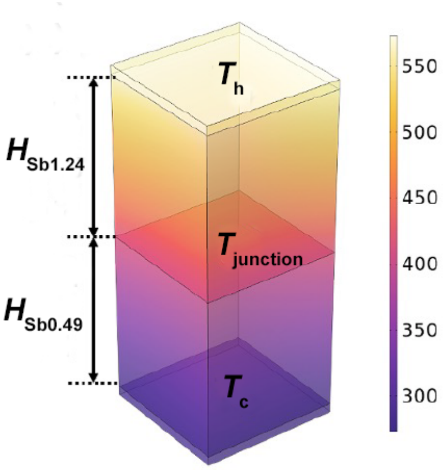

Figure S5. Schematic diagram of segmental simulation of n-type TE leg.

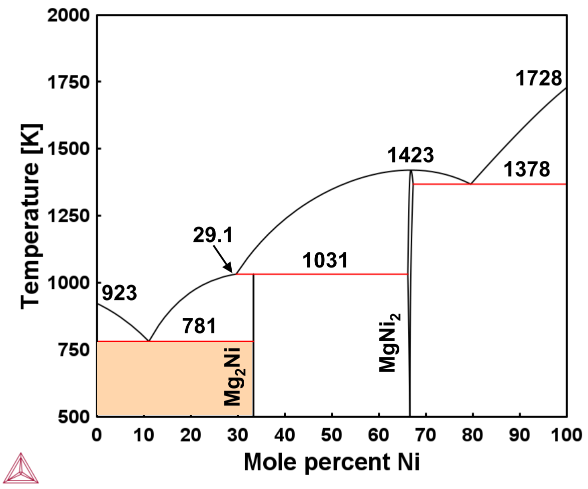

Figure S6. Mg-Ni phase diagram[7]. The common Mg-Ni compounds (Mg_2Ni and $MgNi_2$) and their corresponding melting points.

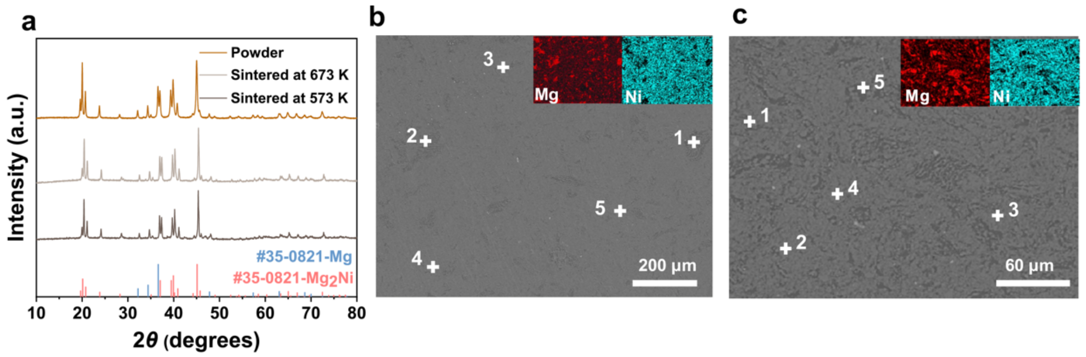

Figure S7. (a) XRD patterns of Mg_2Ni sintered at 573 K and 673 K. SEM image of Mg_2Ni sintered at (b) 573 K and (c) 673 K.

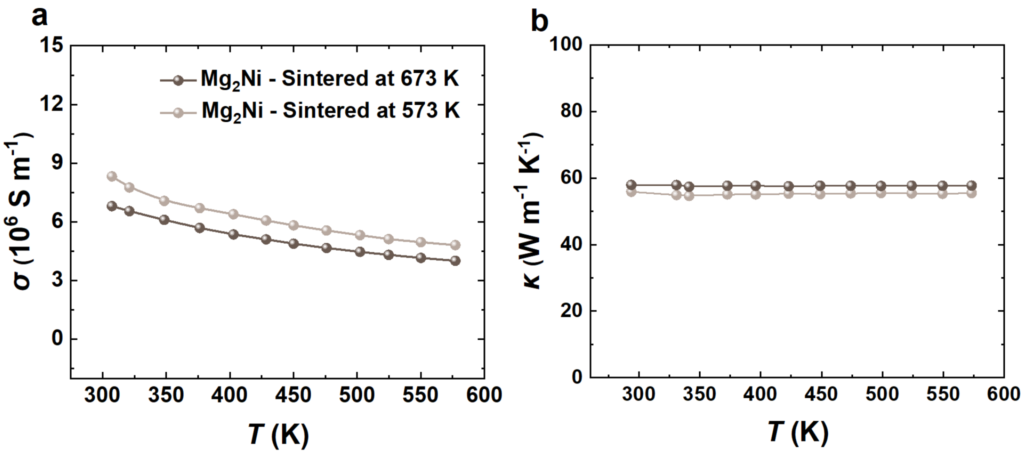

Figure S8. Temperature-dependent (a) electrical conductivity, (b) total thermal conductivity of Mg_2Ni sintered at 573 K and 673 K.

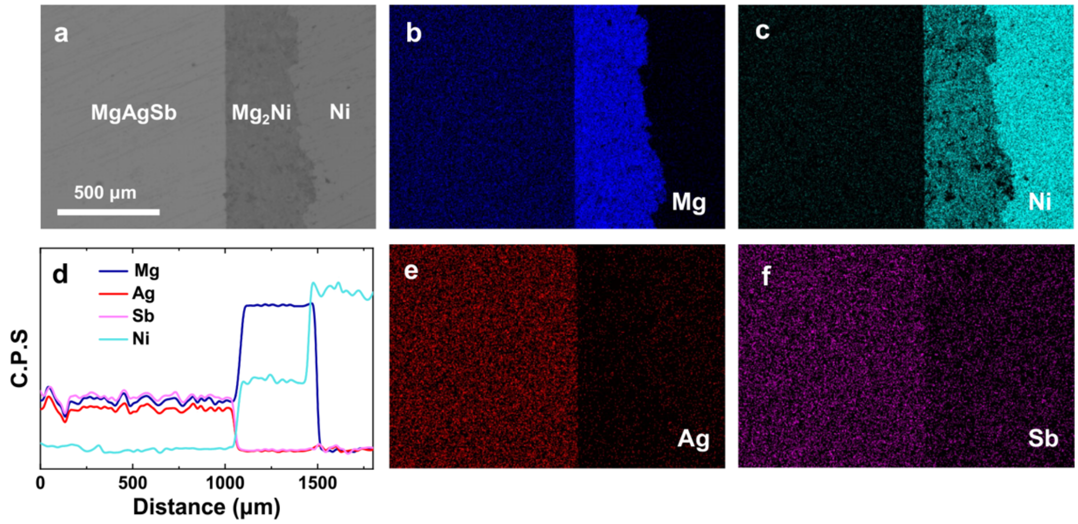

Figure S9. (a) SEM image of as-sintered Ni/Mg_2Ni/MgAgSb junction. (b-c) EDS mapping of Mg and Ni elements. (d) EDS line-scan results of as-sintered Ni/Mg_2Ni/MgAgSb junction. (e-f) EDS mapping of Ag and Sb elements.

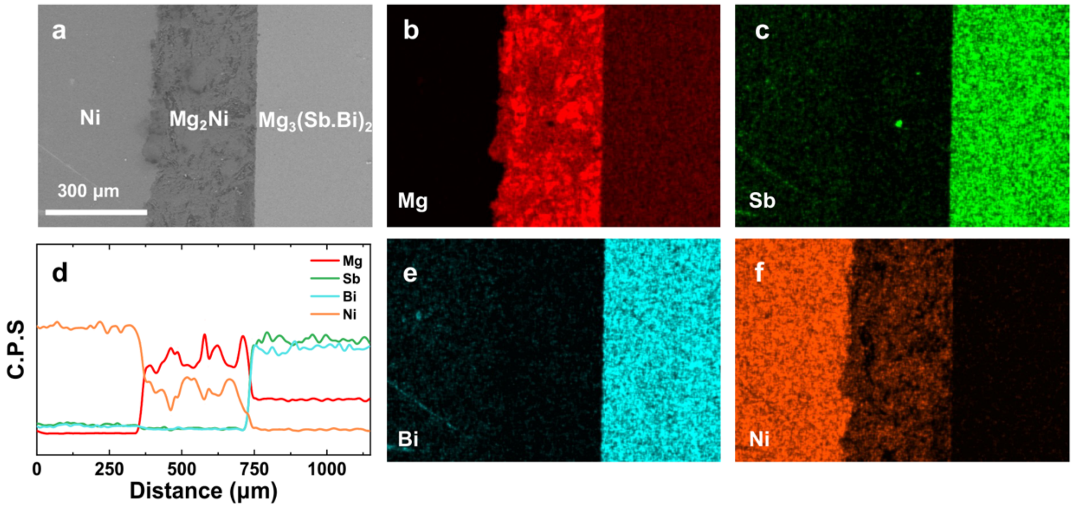

Figure S10. (a) SEM image of as-sintered Ni/Mg_2Ni/ $Mg_3(Sb, Bi)_2$ junction. (b-c) EDS mapping of Mg and Sb elements. (d) EDS line-scan results of as-sintered Ni/Mg_2Ni/ $Mg_3(Sb, Bi)_2$ junction. (e-f) EDS mapping of Bi and Ni elements.

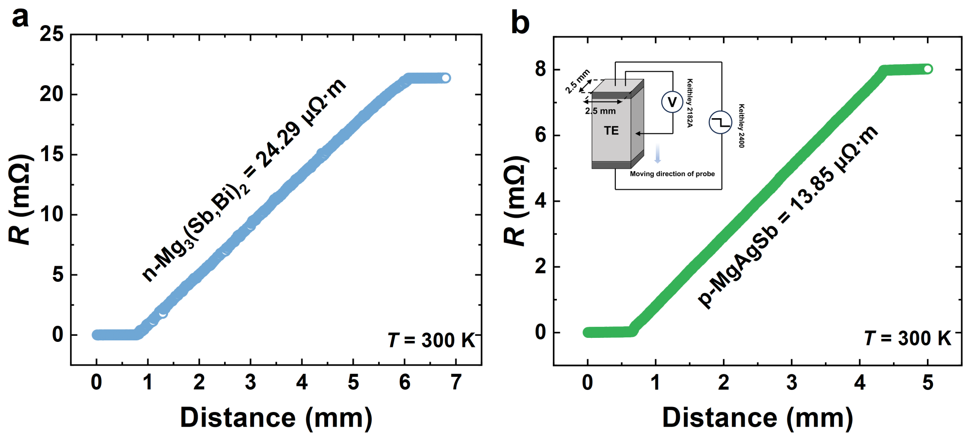

Figure S11. Resistance scanning results of (a) n-type $Mg_{3.15}Mn_{0.05}Sb_{1.24}Bi_{0.75}Se_{0.01}$ and (b) p-type $MgAg_{0.965}Ni_{0.005}Sb_{0.99}$ single leg. Inset: schematic diagram of the contact resistivity measurement.

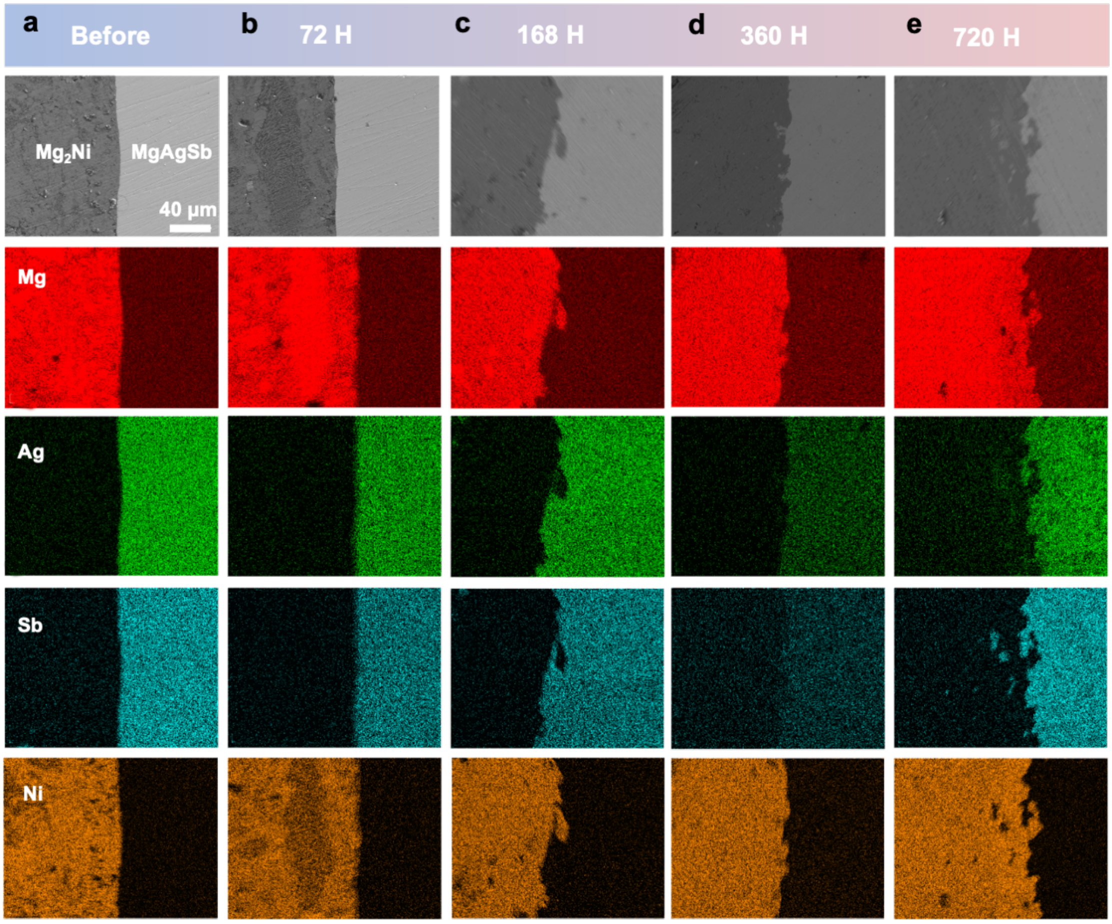

Figure S12. SEM images and EDS mapping results of $Mg_2Ni/MgAg_{0.965}Ni_{0.005}Sb_{0.99}$ junctions. (a) As prepared, and annealing at 573 K for (b) 72 hours, (c) 168 hours, (d) 360 hours, and (e) 720 hours.

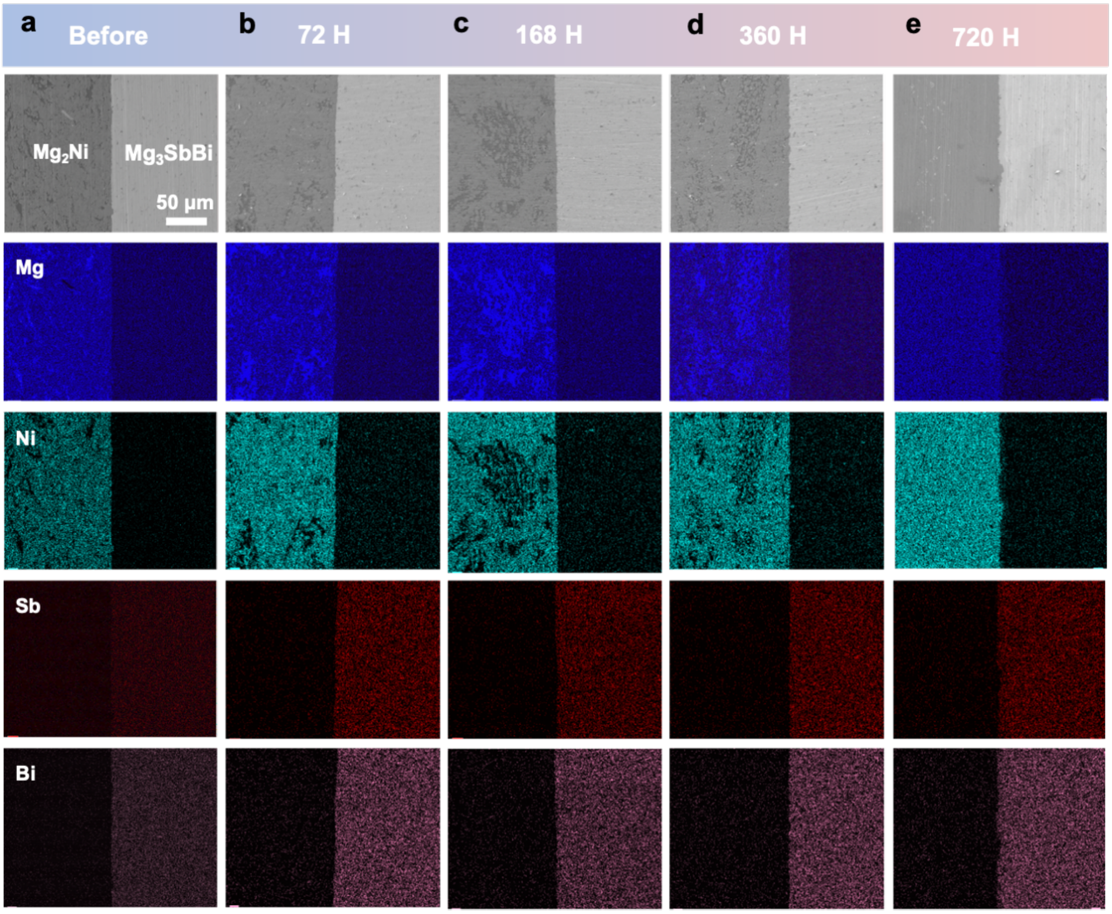

Figure S13. SEM images and EDS mapping results of Mg_2Ni/ $Mg_{3.15}Mn_{0.05}Sb_{1.24}Bi_{0.75}Se_{0.01}$ junctions. (a) As prepared, and annealing at 573 K for (b) 72 hours, (c) 168 hours, (d) 360 hours, and (e) 720 hours.

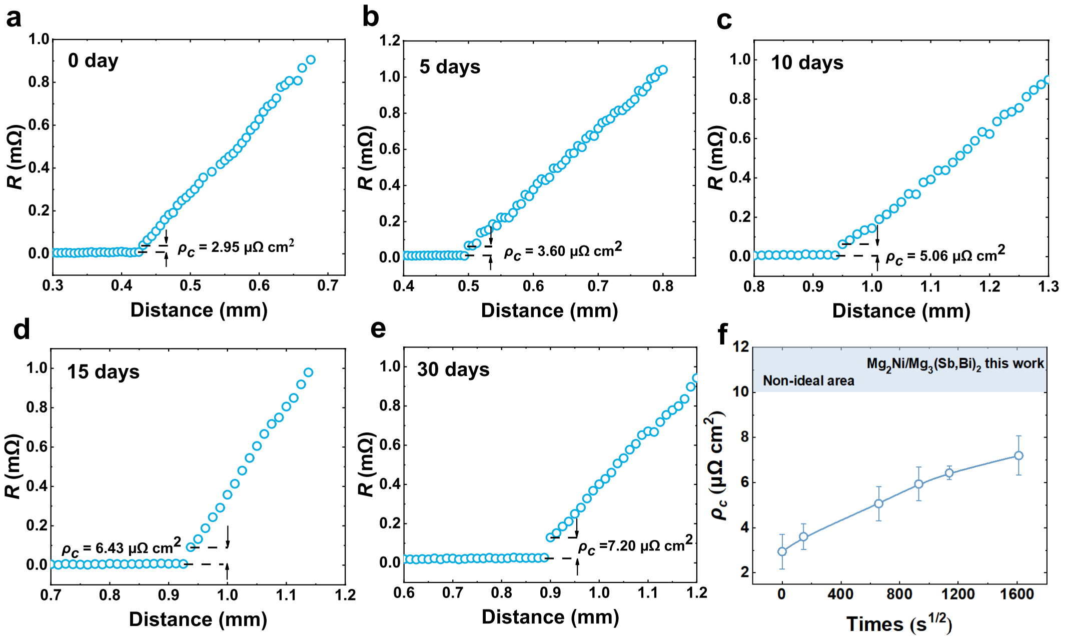

Figure S14. Contact resistivity (ρ_c) of $Mg_2Ni/Mg_{3.15}Mn_{0.05}Sb_{1.24}Bi_{0.75}Se_{0.01}$ junctions with different annealing time at 573 K. (a) 0 day, (b) 5days, (c) 10 days, (d) 15 days, and (e) 30 days. (f) (ρ_c) with the root mean square of aging time at 573 K. Background color in (f) represents the non-ideal range of (ρ_c).

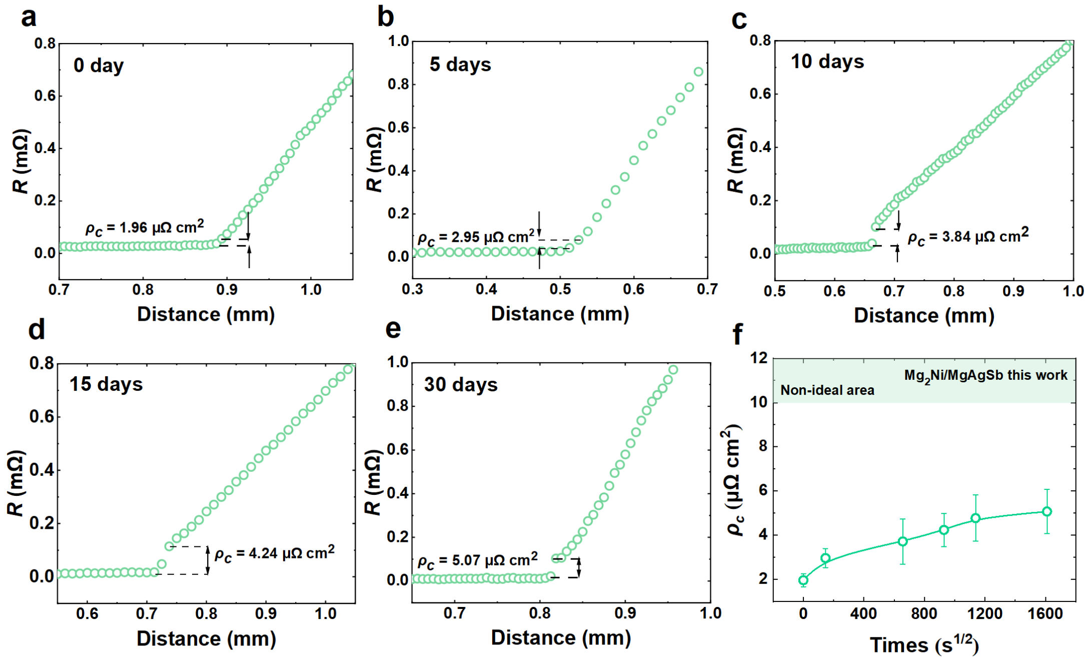

Figure S15. Contact resistivity (ρ_c) of $Mg_2Ni/MgAg_{0.965}Ni_{0.005}Sb_{0.99}$ junctions with different annealing time at 573 K. (a) 0 day, (b) 5days, (c) 10 days, (d) 15 days, and (e) 30 days. (f) (ρ_c) with the root mean square of aging time at 573 K. Background color in (f) represents the non-ideal range of (ρ_c).

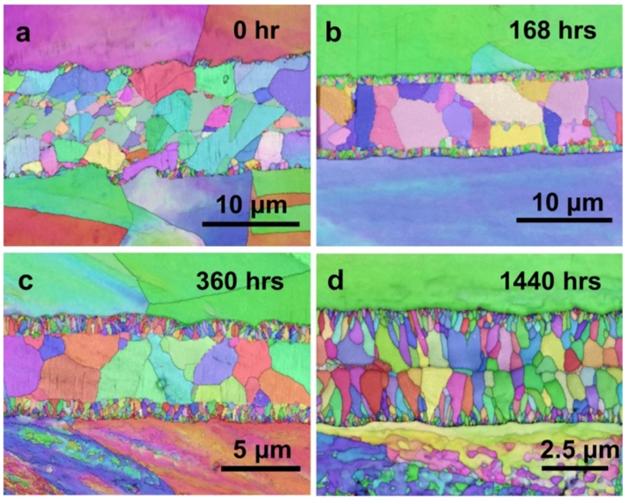

Figure S16. Orientation Mapping of the Ni-Sn IMC joints after annealing for different time. (a) 0 hour, (b) 168 hours, (c) 360 hours, and (d) 1440 hours.

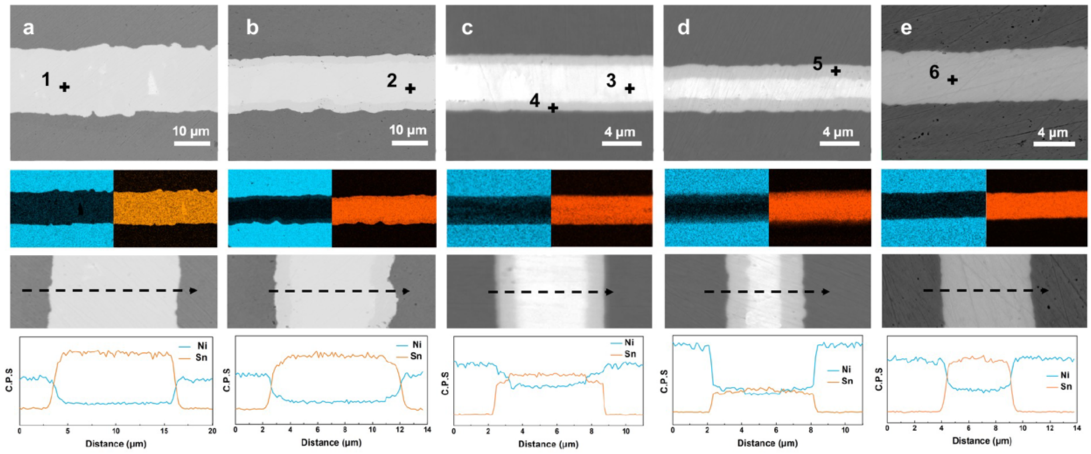

Figure S17. SEM images and EDS mapping results of Ni-Sn IMC joints. (a) As prepared, and annealing at 573 K for (a) 0 hour, (b) 72 hours, (c) 168 hours, (d) 360 hours, and (e) 1440 hours. The EDS results of spectrums in (a-e) are shown in Supplementary Table 3.

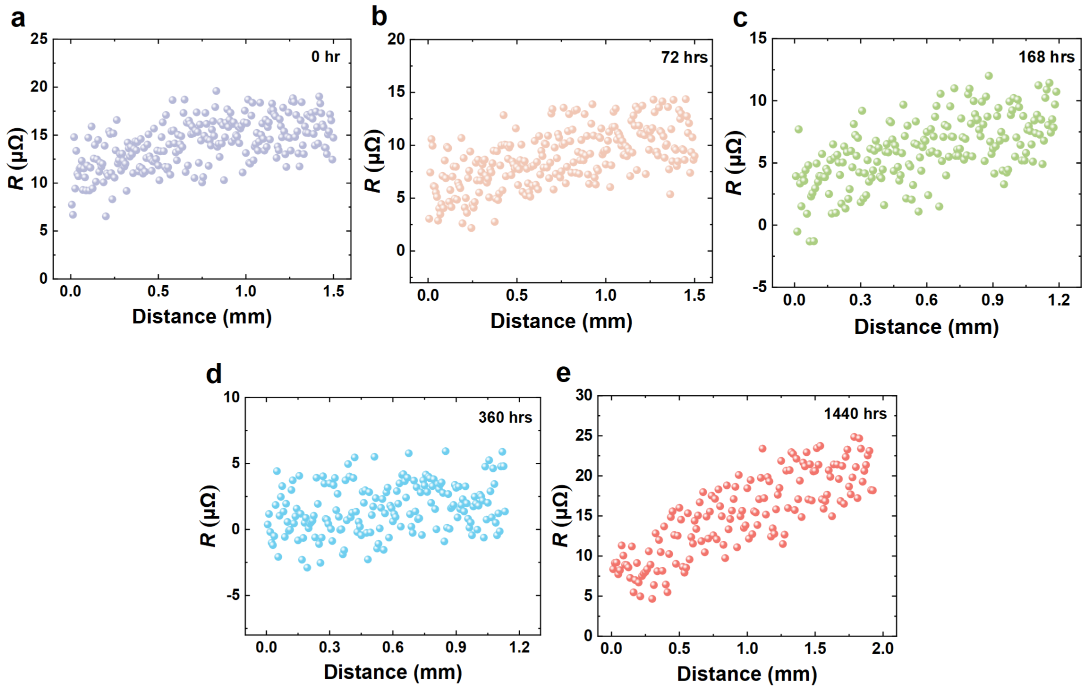

Figure S18. Resistance scanning results of Ni-Sn IMC joints with different annealing time. (a) 0 hours, (b) 72 hours (c) 168 hours, (d) 360 hours, and (e) 1440 hours.

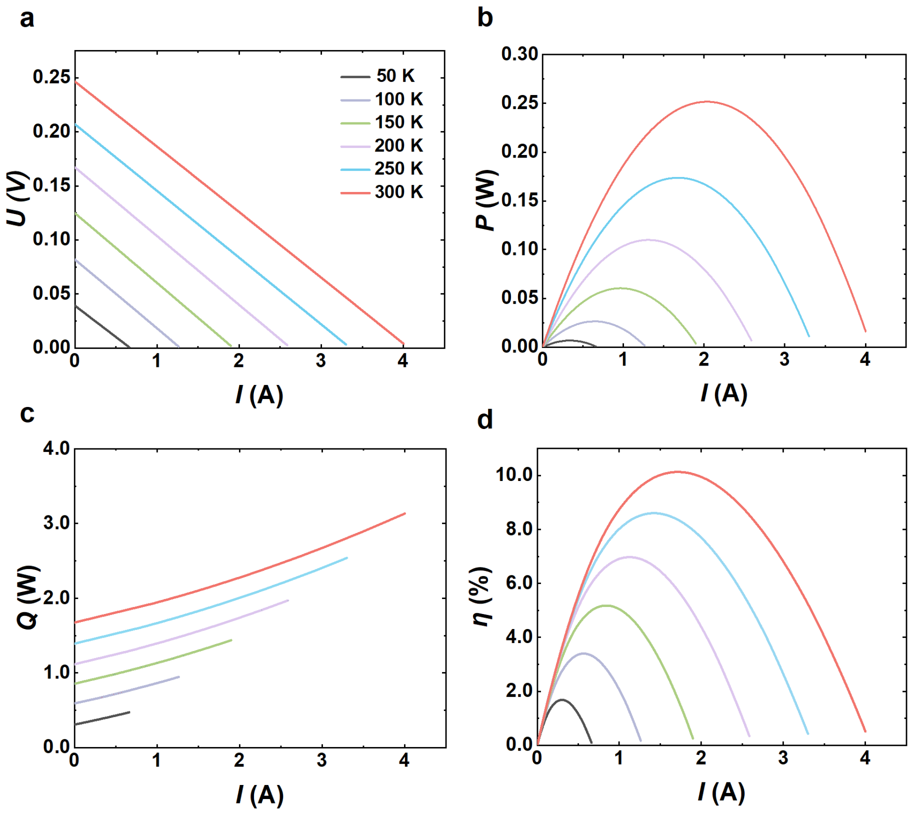

Figure S19. Simulated current-dependent (a) output voltage, (b) output power, (c) heat flow, and (d) efficiency for the single-stage all-Mg-based device under different temperature gradients.

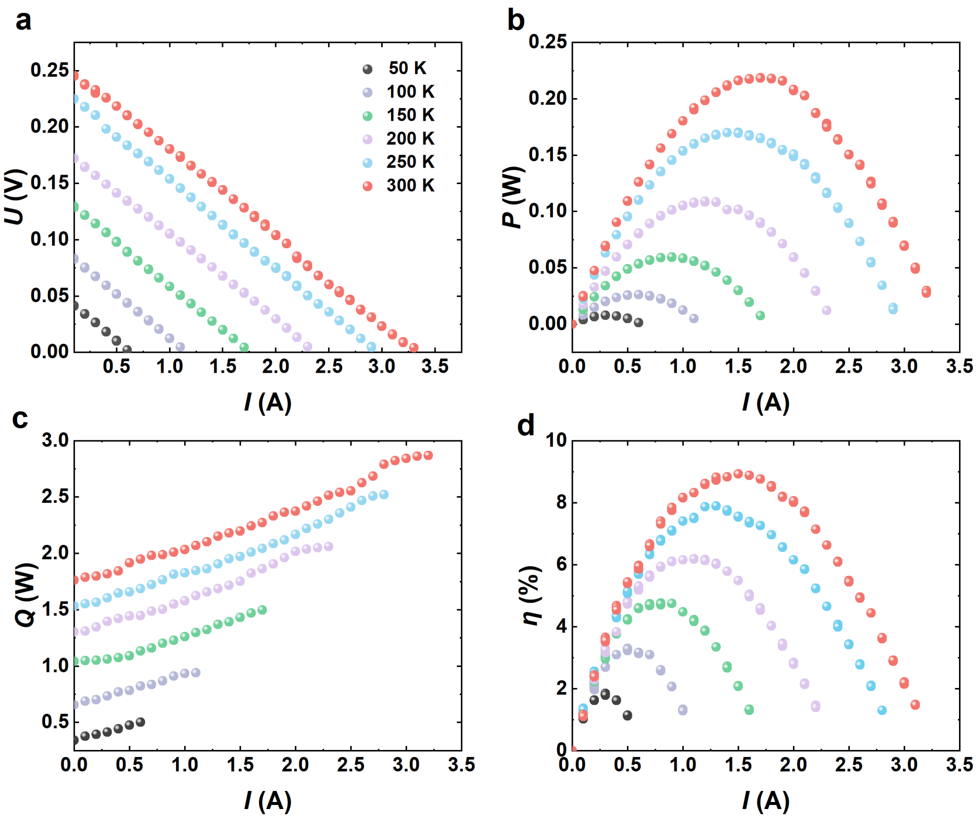

Figure S20. Measured current-dependent (a) output voltage, (b) output power, (c) heat flow, and (d) efficiency for the single-stage all-Mg-based device under different temperature gradients.

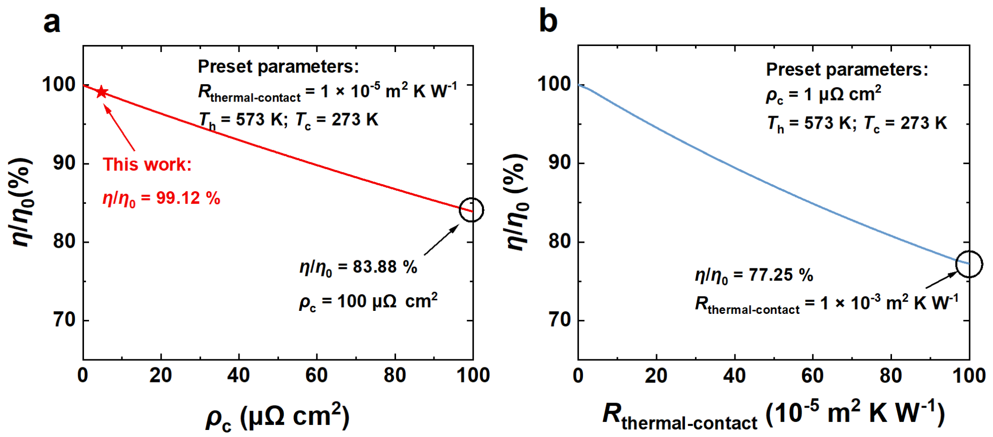

Figure S21. (a) The η/η_0 as a function of the changes in electrical contact resistivity (the thermal contact resistance is fixed at 1×10^{-5} m^2 K W^{-1}). (b) The η/η_0 as a function of the changes in thermal contact resistance (the electrical contact resistivity is fixed at 1 μΩ cm^2).

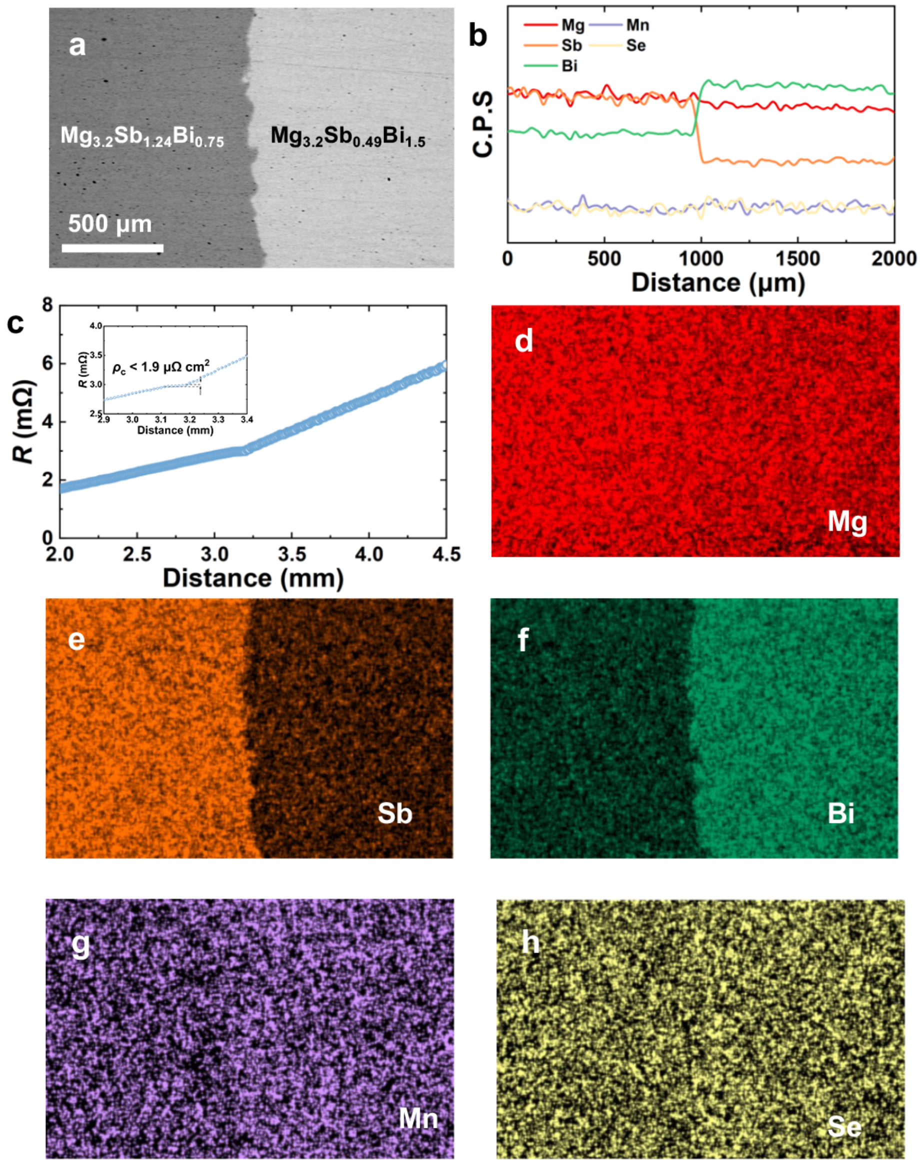

Figure S22. (a) SEM image, (b) EDS line-scan results and (c) resistance scanning results of $Mg_{3.15}Mn_{0.05}Sb_{1.24}Bi_{0.75}Se_{0.01}/Mg_{3.15}Mn_{0.05}Sb_{0.49}Bi_{1.50}Se_{0.01}$ contact interface. Inset: Tested contact resistivity (ρ_c) of $Mg_{3.15}Mn_{0.05}Sb_{1.24}Bi_{0.75}Se_{0.01}/Mg_{3.15}Mn_{0.05}Sb_{0.49}$ -$Bi_{1.5}Se_{0.01}$ junction. (d-h) EDS mapping results of Mg, Sb, Bi, Mn, and Se elements.

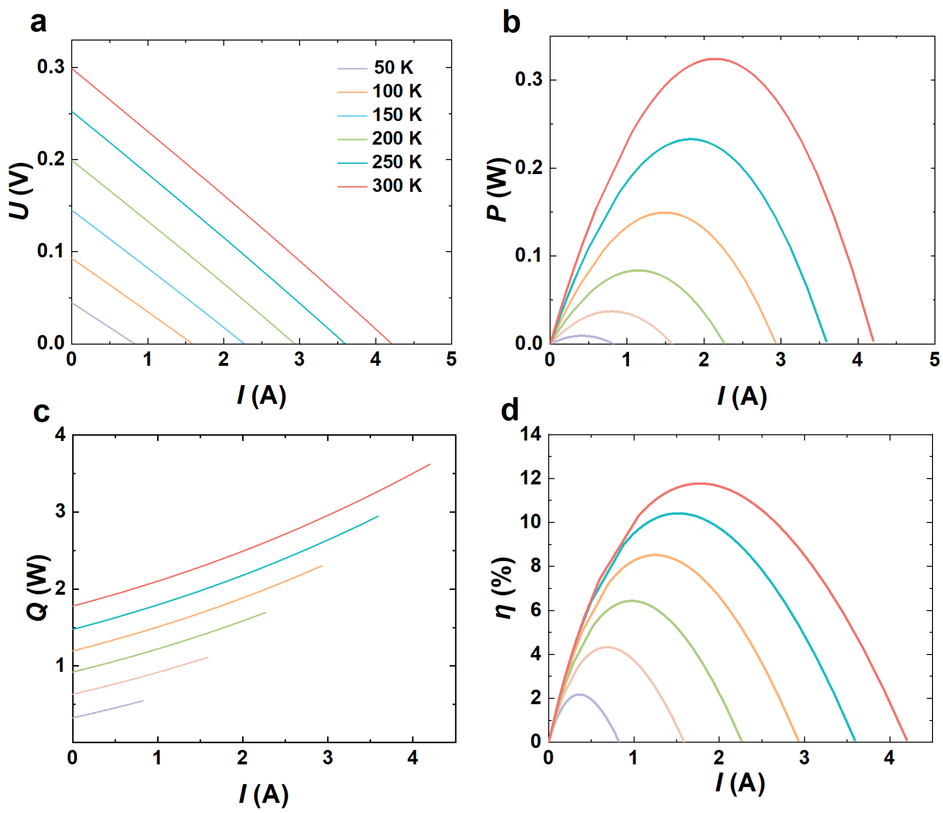

Figure S23. Simulated current-dependent (a) output voltage, (b) output power, (c) heat flow, and (d) efficiency for the segmented all-Mg-based device under different temperature gradients.

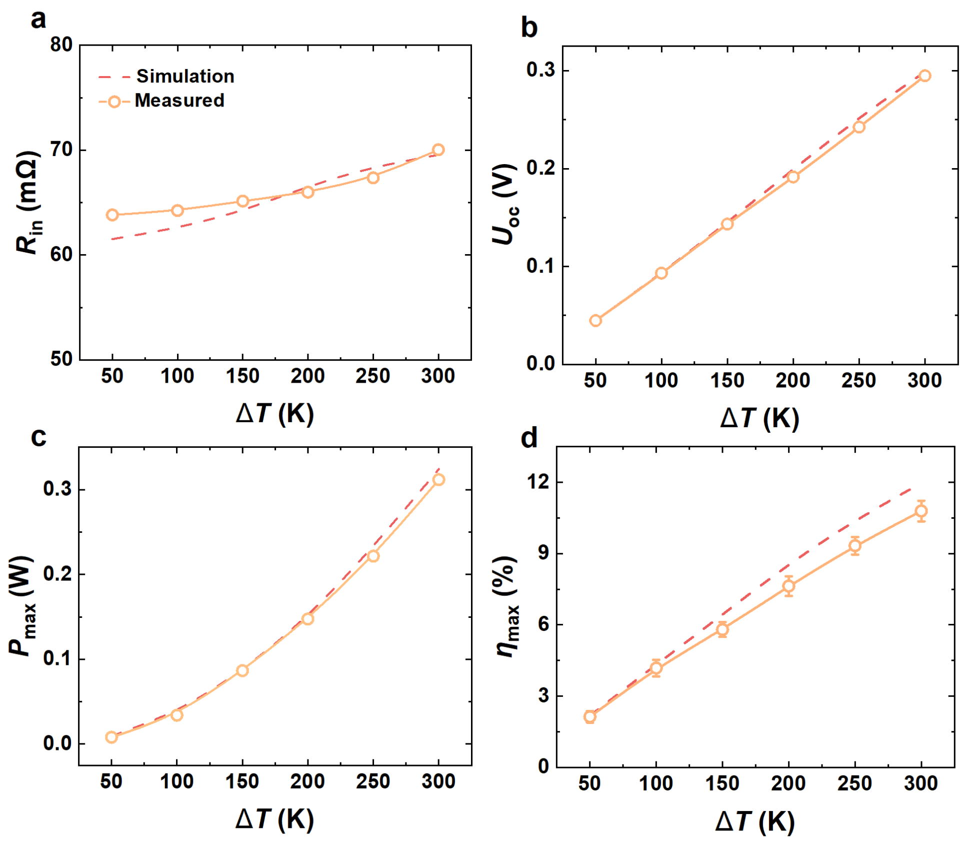

Figure S24. Experimental and simulated temperature difference-dependent (a) resistance (R_{in}), (b) open-circuit voltage (U_{oc}), (c)maximum output power density (P_{max}), and (d) maximum efficiency (η_{max}) for the segmented all-Mg-based device.

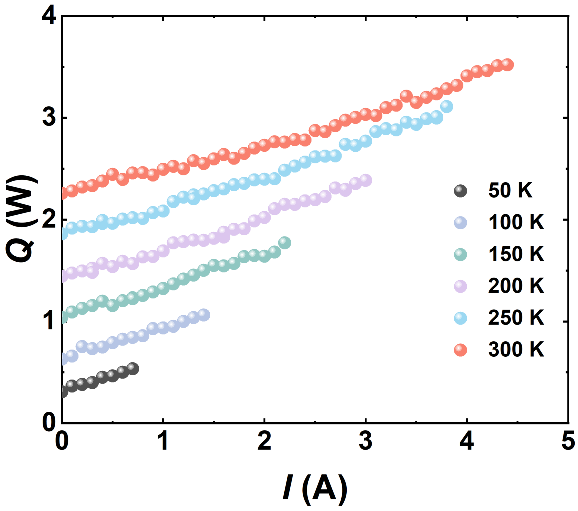

Figure S25. Measured current-dependent heat flow for the segmented all-Mg-based device under different temperature gradients.

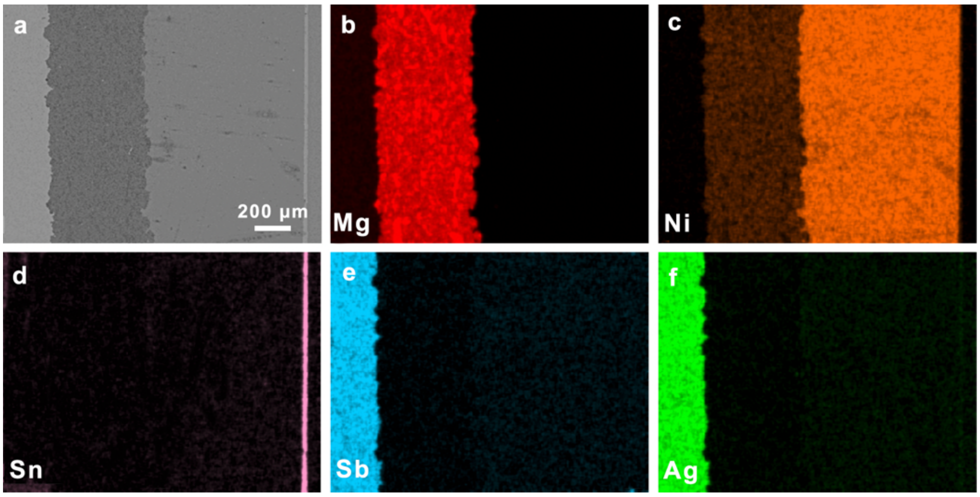

Figure S26. (a) The SEM image of the p-type MgAgSb leg joint after 160-hour aging. (b-f) EDS mapping of Mg, Ni, Sn, Sb and Ag elements.

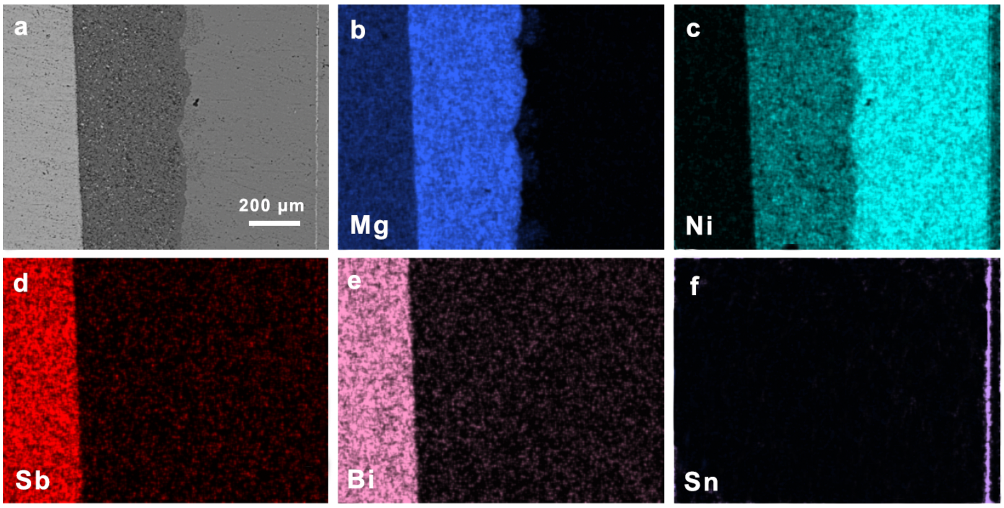

Figure S27. (a) The SEM image of the n-type $Mg_3(Sb, Bi)_2$ leg joint after 160-hour aging. (b-f) EDS mapping of Mg, Ni, Sb, Bi and Sn elements.

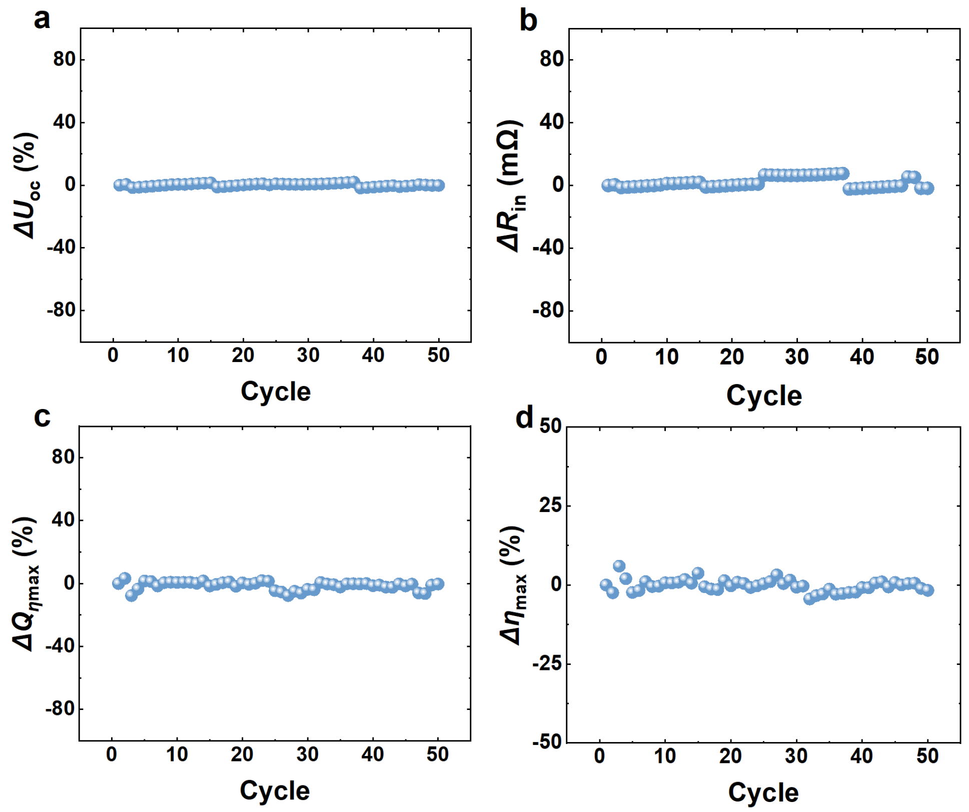

Figure S28. Thermal cycling test results for the segmented all-Mg-based device, showing changes in (a) open-circuit voltage, (b) internal resistance, (c) heat flow at maximum efficiency, and (d) maximum efficiency as a function of thermal cycle number.

Supplementary Table 1 | Finite element simulation setting parameters.

Parameter	Value
Temperature of hot side	573 K
Temperature of cold side	273 K
Thickness of AlN ceramic	0.4 mm
Thickness of nickel electrode	0.2 mm
Electrical contact resistance of p-legs	1.96 μΩ cm^2
Electrical contact resistance of n-legs	2.95 μΩ cm^2
Thermal contact resistance of hot and cold side	1.25×10^{-4} m^2 K W^{-1}
Width of p- and n-legs	3.5mm
Resistance of external load	$R_{in}/10 - 10 \times R_{in}$ (Step: 0.01 Ω)
Length of p- and n-legs	2 mm – 5 mm (Step: 0.2 mm)
Height of p- and n-legs	2 mm – 10 mm (Step: 0.5 mm)

Supplementary Table 2 | The statistical results of the ρ_c for $Mg_2Ni/Mg_3(Sb, Bi)_2$ interfaces with different annealing time.

Annealing time	1	2	3	Mean	Standard Deviation
0	2.69	2.32	3.8	2.93	0.77
3	2.95	3.96	3.89	3.60	0.56
5	4.37	4.95	5.88	5.07	0.76
10	5.98	6.66	5.17	5.94	0.75
15	6.73	6.44	6.12	6.43	0.31
30	7.92	6.23	7.44	7.20	0.88

Supplementary Table 3 | The statistical results of the ρ_c for Mg_2Ni/ MgAgSb interfaces with different annealing time.

Annealing time	1	2	3	Mean	Standard Deviation
0	1.66	1.99	2.25	1.97	0.30
3	2.49	3.32	3.10	2.97	0.43
5	2.95	3.33	4.88	3.72	1.02
10	3.84	3.77	5.11	4.24	0.75
15	4.11	5.98	4.24	4.78	1.13
30	4.55	6.23	4.44	5.07	0.90

Supplementary Table 4 | EDS results of different spectrums in Fig. S17.

Spectrum	Compositions (at.%)		Phase
	Ni	Sn	
1	44.29	55.71	$Ni_{3.1800}Sn_4$
2	44.24	55.76	$Ni_{3.1736}Sn_4$
3	45.31	54.69	$Ni_{3.3139}Sn_4$
4	59.81	40.19	$Ni_{2.9764}Sn_2$
5	61.08	38.92	$Ni_{3.1387}Sn_2$
6	59.99	40.01	$Ni_{2.9988}Sn_2$

Supplementary Table 5 | EDS results of different spectrums in Fig. S7-b.

Specturm	Compoisition (at%)	
	Mg	Ni
1	99.81	3.19
2	66.15	30.85
3	65.73	34.27
4	98.74	1.26
5	97.16	2.84

Supplementary Table 6 | EDS results of different spectrums in Fig. S7-c.

Specturm	Compoisition (at%)	
	Mg	Ni
1	64.51	35.49
2	86.13	13.87
3	59.67	40.33
4	61.87	38.13
5	99.09	0.91

Supplementary Table 7 | Estimated uncertainty of the ΔT_{Cu}, heat flow (Q_{out}), and conversion efficiency (η) of the all-Mg-based module.

**ΔT (K)**	**ΔT_{Cu} (K)**	**$\delta(\Delta T_{Cu})$ (%)**	**$\delta(Q_{out})$ (%)**	**$\delta(\eta)$ (%)**
50	0.11	9.20	11.64	10.56
100	0.23	4.38	8.37	8.04
150	0.37	2.73	7.63	7.11
200	0.46	2.21	7.46	6.82
250	0.60	1.67	7.32	6.62
300	0.69	1.38	7.26	6.47

Uncertainty analysis:

The uncertainty of the temperature difference (ΔT_{Cu}), heat flow (Q_{out}), output power (P), and efficiency (η) was calculated using standard error analysis and propagation methods, as described by the following formulas:

$$\delta(\Delta T_{Cu}) = \sqrt{(T_{Cu1}\times\delta(T_{Cu1}))^2 + (T_{Cu1}\times\delta(T_{Cu1}))^2} / (T_{Cu1} - T_{Cu2}) \quad (1)$$

$$\delta(Q) = \sqrt{\delta(\Delta T_{Cu})^2 + \delta(\kappa)^2 + \delta(A_{Cu})^2 + \delta(L_{Cu})^2} \quad (2)$$

$$\delta(P) = \sqrt{\delta(I)^2 + \delta(U)^2} \quad (3)$$

$$\delta(P+Q) = \sqrt{(P\times\delta(P))^2 + (Q\times\delta(Q))^2} \; / \; (P+Q) \quad (4)$$

$$\delta(\eta) = \sqrt{\delta(P)^2 + \delta(P+Q)^2} \quad (5)$$

$$\delta(\omega) = \sqrt{\delta(P)^2 + \delta(A_{Cu})^2} \quad (6)$$

The uncertainty of κ_{Cu}, A_{Cu}, L_{Cu} (8 mm ± 0.1 mm), I, U, and T are 7%, 0.5%, 1.25%, 1%, 1%, and ±0.01 K, respectively. Table R1 lists the estimated uncertainty of ΔT_{Cu}, Q_{out}, and η measurements for the all-Mg-based segmented module.

Supplementary Reference

1. Pouranvari M, Ghasenu A and Salmasi A. On the inability of the moving interface model to predict isothermal solidification time during transient liquid phase (TLP) bonding of Ni-based superalloys. *Metall Mater Trans A* 2022; **53**: 126–35.
2. Yin L, Li X and Bao X *et al.* CALPHAD accelerated design of advanced full-Zintl thermoelectric device. *Nat Commun* 2024; **15**: 1468.
3. Schmetterer C, flandorfer H and Richter K *et al.* A new investigation of the system Ni-Sn. *Intermetallics* 2007; **15**: 869-84.
4. Cheng J, Xue W and Zhang T *et al.* A universal approach to high-performance thermoelectric module design for power generation. *Joule* 2025; **9**:101818.
5. Okamoto H. Ni-Sn (Nickel-Tin). *J Phase Equilibria Diffus* 2008; **29**: 297–8.
6. Liu Z, Mao J and Sui J *et al.* High thermoelectric performance of α-MgAgSb for power generation. *Energy Environ Sci* 2018; **11**: 23–44.
7. Okamoto, H. Mg-Ni (Magnesium-Nickel). *J Phase Equilibria Diffus* 2007; **28**: 303.
